# White-rot basidiomycetes *Junghuhnia nitida* and *Steccherinum bourdotii*: Oxidative potential and laccase properties in comparison with *Trametes hirsuta* and *Coriolopsis caperata*

**DOI:** 10.1371/journal.pone.0197667

**Published:** 2018-06-01

**Authors:** Olga A. Glazunova, Natalia V. Shakhova, Nadezhda V. Psurtseva, Konstantin V. Moiseenko, Sergei Y. Kleimenov, Tatiana V. Fedorova

**Affiliations:** 1 A.N. Bach Institute of Biochemistry, Research Center of Biotechnology of the Russian Academy of Sciences, Moscow, Russia; 2 Komarov Botanical Institute of the Russian Academy of Sciences, St. Petersburg, Russia; 3 Koltzov Institute of Developmental Biology of the Russian Academy of Sciences, Moscow, Russia; USDA Forest Service, UNITED STATES

## Abstract

White-rot basidiomycetes from the poorly studied residual polyporoid clade of Polyporales order *Junghuhnia nitida* (Pers.) Ryvarden and *Steccherinum bourdotii* Saliba & A. David grow as secondary xylotrohps on well decomposed woody materials. The main objective of the current study was to compare oxidative potential, growth, production of oxidative enzymes and laccase properties of *J*. *nitida* and *S*. *bourdotii* with that of typical primary xylotrohps *Trametes hirsuta* (Wulfen) Lloyd and *Coriolopsis caperata* (Berk.) Murrill, belonging to the core polyporoid clade. For the first time we report species *J*. *nitida* and *S*. *bourdotii* as active laccase producers. New laccases from *J*. *nitida* and *S*. *bourdotii* were purified and characterized. They had an identical molecular weight of 63 kDa and isoelectric points of 3.4 and 3.1, respectively. However, the redox potential of the T1 copper site for both *J*. *nitida* (610 mV) and *S*. *bourdotii* (640 mV) laccases was lower than those for *T*. *hirsuta* and *C*. *caperata* laccases. The new laccases showed higher temperature optima and better thermal stability than *T*. *hirsuta* and *C*. *caperata* laccases. Their half-lives were more than 40 min at 70 °C. The laccases from *J*. *nitida* and *S*. *bourdotii* showed higher affinity to syringyl-type phenolic compounds than *T*. *hirsuta* and *C*. *caperata* laccases. The oxidative potential of studied fungi as well as the properties of their laccases are discussed in terms of the fungal life-style.

## Introduction

White-rot fungi are wood degraders that play a very important role in the forest ecosystems. A distinct feature of this fungal group is their ability to decompose all plant cell wall components including lignin up to the formation of carbon dioxide and water. The process of lignocellulose substrate degradation by these fungi comprise a complicated network of enzymatic reactions catalyzed by cellulases, various peroxidases (e.g. manganese and lignin peroxidases), laccases and other enzymes [1,2]. It was shown that the composition of lignin-modifying enzymes of the white-rot fungi reflects their life-style and trophic specialization [3–6].

Many of the typical white-rot basidiomycetes are represented by the members of the order Polyporales which, according to Binder and coworkers [7], can be divided into four clades: the core polyporoid clade, the phlebioid clade, the antrodia clade and the residual polyporoid clade. While the white-rot basidiomycetes from the core polyporoid (e.g. genera *Coriolopsis*, *Lentinus*, *Trametes*, *Ganoderma*) and phleboid (e.g. genera *Phlebia*, *Phanerochaete*, *Bjerkandera*) clades are studied relatively well, information about the fungi from the residual polyporoid and antrodia clade is rather limited.

Some representatives of the residual polyporoid clade are typical secondary xylotrophs colonizing well decomposed woody materials. It has been suggested that saprotrophic fungi from the later successional stages have higher laccase activity than those from the earlier stages [8]. At least three species of the *Steccherinaceae* family *Steccherinum ochraceum* [9,10], *Steccherinum murashkinskyi* LE-BIN 1963 [11], and *Antrodiella faginea* LE-BIN 1998 [12] were previously found as promising laccase producers.

Laccases (EC 1.10.3.2, benzenediol:oxygen oxidoreductase) belong to a family of multi-copper oxidases. They can oxidize a broad range of compounds including substituted phenols, arylamines and aromatic thiols to the corresponding radicals with concomitant four-electron reduction of the molecular oxygen to water without releasing toxic peroxide intermediates. The exact physiological role of laccases in fungi is not clear. Laccases could be involved in morphogenesis, fungal plant-pathogen/host interaction, stress defense, pigment formation, detoxification of phenolic compounds and lignin degradation [13–16].

After the discovery of the first fungal laccase in 1896 [17] more than 100 laccases from different fungi were isolated and characterized [14,18,19]. The biochemical properties of laccases vary significantly. In particular, the substrate specificity of the enzymes is different depending on the source of laccase [14,20].

The current investigation focuses on the two strains of the secondary xylotrohpic basidiomycetes *Steccherinum bourdotii* Saliba & A. David and *Junghuhnia nitida* (Pers.) Ryvarden, that both are representatives of the residual polyporoid clade. The goal of the study was to compare their oxidative potential, growth and production of oxidative enzymes with typical primary xylotrohps *Trametes hirsuta* (Wulfen) Lloyd and *Coriolopsis caperata* (Berk.) Murrill, belonging to the core polyporoid clade. The laccases produced by the studied fungi were comprehensively investigated and their substrate specificity was discussed from the perspective of the fungal wood colonization strategies.

## Materials and methods

### Strains description

*Junghuhnia nitida* strain was obtained in 2005 from basidiospores of basidioma grown on decomposed wood and old fruit bodies of *Trametes* sp.; Russia, Far East, Primorsky territory, Kedrovaya Pad nature reserve, near Kedrovaya river, 43°06′32^2^ N, 131°32′07^2^ E.

*Steccherinum bourdotii* strain was obtained in 1986, Russia, Western Caucasus; the strain was received in 2010 from R. H. Petersen (CullTenn 8917, UT, Knoxville, USA).

Both strains were deposited in the Komarov Botanical Institute Basidiomycetes Culture Collection (LE-BIN) under the identifiers LE-BIN 2013 for *J*. *nitida* and LE-BIN 2738 for *S*. *bourdotii*. In the collection the strains are maintained in culture tubes on agar slants and in 2 mL microvials in distilled water at 4 °C, and in 2 mL cryovials in 10% glycerol at –80 °C.

### General cultural studies

For the macro- and micromorphological study inoculum plugs (6 mm in diameter) were placed mycelium side down on the edge of 90 mm Petri plates containing BWA medium (beer-wort 4%, Northern Brewery, Saint-Petersburg, Russia; agar 20 g·l^-1^, Difco, USA). Three replicates of each strain were incubated for 4 weeks in the growth chamber at 25 °C in the dark. The growth rate was recorded daily. The advancing zone was studied at week 2 and colony morphologies–at week 4. The micromorphology was studied at weeks 2 and 4 under Zeiss Axio Scope A1 (Zeiss, Germany) using transmitted light. Morphological description was done using traditional terminology [21].

Induction of fruiting body formation was performed on birch sawdust:wheat bran (3:1 respectively) which were mixed with boiling water until the substrate was damp. For inoculating the substrate, the strains were grown in 60 mm Petri plates on BWA medium for 2 weeks. The colonies were diced under sterile conditions, added to the substrate in beakers and incubated at room temperature for 3 weeks in the dark, with subsequent exposure to natural light for fructification.

For a qualitative assessment of the overall oxidoreductase activity, the strains were grown in the dark at 25 °C in 90 mm Petri plates on BWA medium. The inoculation was done as described above using 14-day old mycelium. The activity was assayed weekly during 4 weeks of incubation. Mycelial blocks of 6 mm in diameter were cut near the edge of a growing colony and tested by express method using 1.0% syringaldazine (Sigma, USA) in C_2_H_5_OH and 2.0% guaiacol (Sigma, USA) in Н_2_О as substrates. The activity was evaluated visually by the color reaction intensity on a scale from “–" (no activity) to"+++" (the highest activity) at 5, 15, 30, 60 min, 3 and 24 h [11].

**Fig 1:**
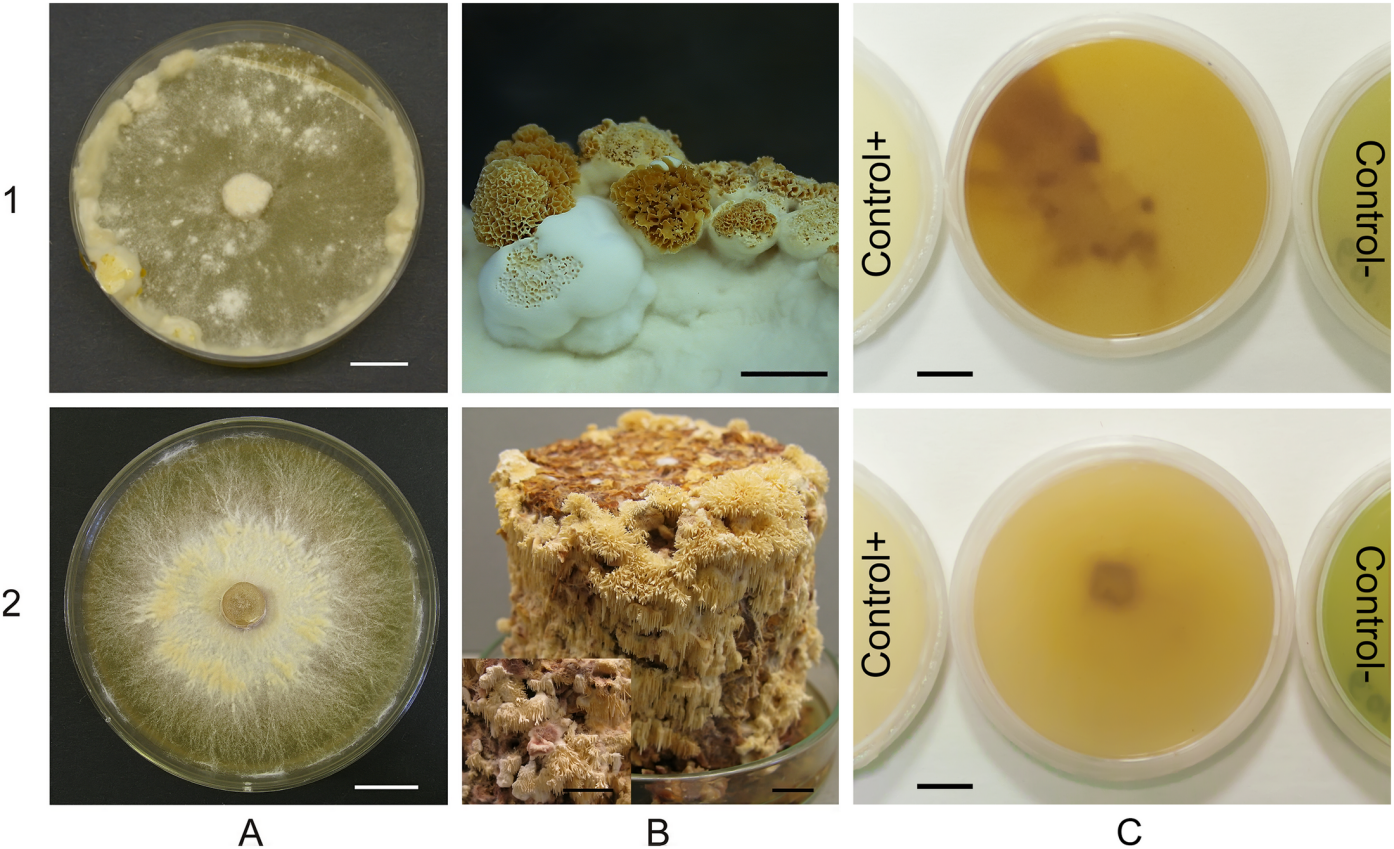
Macro–, fruiting-bodies morphology and Azure B decolorization of *Junghuhnia nitida* LE-BIN 2013 (1) and *Steccherinum bourdotii* LE-BIN 2738 (2): colonies on BWA medium (A); fruiting bodies on sawdust substrate (B); decolorization of Azure B in MEA medium (C). Bars –10 mm. Control–: plate without fungus. Control+: plate with *Trametes hirsuta* LE-BIN 072.

For a qualitative assessment of the lignin peroxidase activity the strains were grown in the dark at 25 °C in 90 mm Petri plates on MEA medium containing synthetic dye Azure B (Sigma, USA) with concentration of 75 mg·l^-1^. The radial mycelial growth and the decolorized area were examined and the decolorization ring diameter measured [3]. The activity was evaluated by the intensity of agar-plates dye decolorization on a scale from "–" (no activity) to"+++" (the highest activity) within 4 weeks of incubation (Fig 1C).

### Cultivation of the fungal strains in liquid medium in the presence of lignocellulose

The inoculum of *J*. *nitida* and *S*. *bourdotii* was grown stationary in 750 ml Erlenmeyer flasks with porcelain beads using glucose-peptone medium of the following composition (g·l^-1^): glucose– 10.0, peptone– 3.0, KH_2_PO_4_ − 0.6, K_2_HPO_4_ − 0.4, ZnSO_4_x7H_2_O – 0.001, FeSO_4_x7H_2_O – 0.0005, MnSO_4_,– 0.05, MgSO_4_x7H_2_O – 0.5, CaCl_2_ − 0.5, H_2_O, pH– 6.0 (before sterilization). The inoculated flasks were incubated at 25 C in the dark for 15–20 days. When the surface of the medium in the flasks was covered by mycelium the flasks were shaken manually until porcelain beads crushed the mycelium into a homogeneous suspension. The inoculum was added to the 750 ml Erlenmeyer flasks contained glucose-peptone medium (GP medium) supplemented with lignocellulose (LC medium; 50 g·l^-1^ of milled wood sawdust d ≥1 mm in GP medium separated from the fungus by a nylon mesh) [22]. Static surface cultivation (without shaking) was carried out at 25 °C for 27 days in the dark. Cultural liquids were used for determination of oxidase activities.

The laccase activity was measured by monitoring of absorbance decrease at 525 nm using syringaldazine (ε_520_ = 65,000 M^−1^ cm^−1^) as a chromogenic substrate. The enzymatic reaction was carried out for 3 min in 2 ml reaction mixtures containing 0.1 M sodium acetate buffer, pH 4.5, 0.42 mM syringaldazine, and the required amount of the cultural liquids to determine initial rates of the substrate oxidation [23].

The manganese peroxidase (MnP) activity was determined directly by formation of Mn^3+^- tartrate complex (ε_238_ = 6500 M^−1^cm^−1^) during oxidation of MnSO_4_. 2 ml reaction mixtures contained 0.1 mM MnSO_4_ in 0.1 M sodium tartrate, pH 4.5 and 0.1 mM H_2_O_2_. The initial rates of reaction were measured by recording linear increase of absorbance at 238 nm for 3 min [24].

The Mn^2+^- independent peroxidase (versatile peroxidase, VP or lignin peroxidase, LiP) activity was evaluated by formation of the veratraldehyde (ε_310_ = 9300 M^−1^ cm^−1^) from veratryl alcohol (3,4-dimethoxybenzyl alcohol)[25]. The enzymatic reaction was assayed in 2 ml mixtures containing 10 mM veratryl alcohol, 0.1 M sodium tartrate, and 5 mM H_2_O_2_, and culture supernatant at pH 4.5, and the change of optical density was recorded at 310 nm for 3 min.

All measurements of enzymatic activities were carried out at +25 °C in the thermostated cell of Perkin Elmer Lambda 35 spectrophotometer (USA) with Peltier Temperature Controller. One unit of activity was defined as (μM of product formed) × min^-1^ × dilution. All assays were performed in triplicate.

### DNA extraction, PCR amplification and phylogenetic analysis

For nucleic acid extraction, fungal mycelium was ground in liquid nitrogen. Total DNA extraction was performed using DNeasy Plant Mini Kit (Qiagen, US).

Partial 18S, 28S and complete 5.8S rRNA nucleotide sequences, as well as ITS1 and ITS2 sequences were PCR amplified using standard oligonucleotide primers to the conserved regions of the ribosomal genes: ITS1F 5'–CTT GGT CAT TTA GAG GAA GTA A–3' and ITS4B 5'–CAG GAG ACT TGT ACA CGG TCC AG–3'. PCR amplification was performed using the Encyclo PCR kit (Evrogen, Russia) under the following conditions: 1 cycle of 5 min at 95 °C; 25 cycles of 1 min at 90 °C, 1 min at 56 °C, and 1 min at 72 °C); 1 cycle of 10 min at 72 °C. PCR products were purified from agarose gel by a QIAquick Gel Extraction Kit (Qiagen, USA) and sequenced using the standard Sanger sequencing method. Obtained sequences were submitted to the GenBank with the accession numbers KY095106 (*J*. *nitida*) and KY095107 (*S*. *bourdotii*).

**Fig 2:**
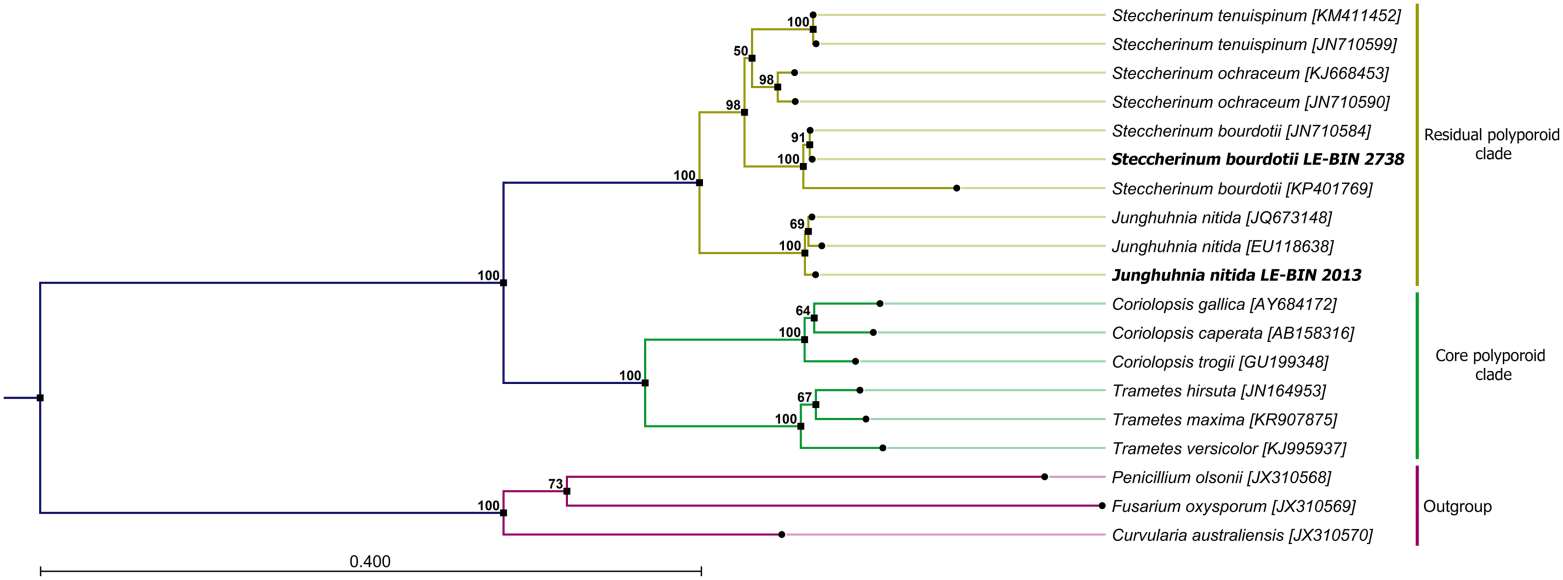
Maximum likelihood (ML) phylogenetic tree of ribosomal gene sequences. Numbers at nodes are bootstrap percentages from 100 replicates. Fungi from the residual and the core polyporoid clades are highlighted in yellow and green respectively. Three ascomycete sequences used as the outgroup are highlighted in violet.

Multiple sequence alignment of the obtained sequences with 102 relevant nucleotide sequences from the GenBank was generated with the ClustalW program [26]. The best fitting model of sequence evolution (GTR) was determined using jModelTest2 software [27] with 11 substitution schemes. The phylogenetic tree was constructed using the PhyML program (v3.0) [28] (Figure A in S1 File). The reliability for the internal nodes was assessed using the bootstrapping method (100 bootstrap replicates). The representative subtree (Fig 2) was extracted from the original one using APE package [29] for R. The GeneBanck identifiers for all additional sequences were placed at the corresponding leaves of phylogenetic trees (Figure A in S1 File and Fig 2).

### Laccase identification and purification

The fungal inoculum was prepared as described previously (see "Cultivation of the fungal strains in liquid medium in the presence of lignocellulose"). The inoculum was added to the 750 ml Erlenmeyer flasks contained glucose-peptone medium supplemented with 0.15 g·l^-1^ CuSO_4_ for *J*. *nitida* and *S*. *bourdotii* and 0.25 g·l^-1^ for *T*. *hirsuta* and *C*. *caperata* to a final volume of 10%. Submerged cultivation was carried out on a circular shaker (180 rpm) at 27 °C for 25 days in the dark. Laccase activity was measured daily by sampling of the culture liquid. For *J*. *nitida* and *S*. *bourdotii* the culture broth was harvested by paper filtration after 20 days of the cultivation, and for *T*. *hirsuta* and *C*. *caperata*—after 10–12 days of the cultivation.

Laccase activity was determined spectrophotometrically using 10 mM pyrocatechol (Sigma, USA), in 0.1 M Na-acetate buffer at pH 4.5 as a chromogenic substrate [30]. An increase in absorbance at 410 nm (ε = 740 M^-1^ cm^-1^) was monitored for 3 min. One unit of activity was defined as μM of product formed per min by 1 μg·l^−1^ of enzyme. The rates of enzymatic reactions were recorded using Perkin Elmer Lambda 35 spectrophotometer (USA).

First stages of laccase purification from culture broth after submerged cultivation was performed according to [31]. To purify the laccases to a homogeneous state an additional FPLC step was performed on a Superdex 75G (26/60) column (GE Healthcare Life Sciences, USA) equilibrated with 20 mM potassium phosphate buffer pH 6.5.

The identification of *J*. *nitida* (JnL) and *S*. *bourdotii* (SbL) laccases by the MALDI-TOF/TOF MS/MS was performed by cutting the proteins bands obtained after the SDS-PAGE analysis. The gel samples of the native and deglycosylated (see below) laccases were digested with the trypsin, and the resulting peptides were spotted on a MALDI target plate. The peptides were analyzed by the MALDI-TOF/TOF spectrometry on a Bruker Ultraflex II MALDI-TOF/TOF mass spectrometer (Germany) equipped with a UV laser (Nd) in positive ion regimen with a reflectron for peptide fingerprinting. The fragmentation spectra were obtained using a tandem regimen of the device, and the accuracy of measurement of fragmented ions was no less than 1 Da. The mass spectra were processed using the FlexAnalysis 3.3 program (Bruker Daltonics, Germany). Sequences of the peptides individually derived from the fragmentation data were analyzed by BLASTp (http://blast.ncbi.nlm.nih.gov/Blast.cgi).

### Laccase characterization

The molecular weights of the laccases were determined by the SDS–PAGE according to Laemmli [32] in a Mini-PROTEAN 3 device (Bio-Rad, USA). The protein bands were stained with Coomassie Brilliant Blue R-250 (Sigma, USA). A PageRuler Prestained Protein Ladder (Fermentas, Lithuania) with a range of 10–200 kDa was used as a standard. The analytical isoelectric focusing on polyacrylamide gel (IEF-PAGE) was carried out using Ampholyte 3/5 (Bio-Rad, USA) on a Mini IEF Cell (Bio-Rad, USA). The protein mixture from IEF Calibration Kit Low-Range p*I* (pH 2.5–6.5) (Amersham, USA) was used as a standard.

The laccases were deglycosylated by treatment with PNGase F (Sigma, USA). The glycoproteins were denatured for 10 min at 100 °C in denaturing buffer (1.86% SDS and 5% β-mercaptoethanol) and then incubated for 90 min at 37 °C with PNGase F in 0.1 M citrate-phosphate buffer pH 5.5 according to manufacturer’s instructions. Deglycosylation was confirmed by SDS-PAGE.

The UV-visible absorption spectra of the laccases (2.0 mg·ml^-1^) in 50 mM potassium phosphate buffer pH 6.5 were recorded with a PerkinElmer Lambda 25 spectrophotometer (USA) in a 1 cm quartz cell at 25 °C in the range of 200–800 nm.

The redox potential (*E*^0^) of the laccase T1 copper site was determined by the anaerobic redox titration technique [33]. The Na_2_(IrCl_6_) and K_4_[Fe(CN)_6_] were used as a redox pair.

The melting points *T*_max_ of the laccases were determined by differential scanning calorimetry (DSC) using Nano Differential Scanning Calorimeter (N-DSC III) (TA Instruments, USA) at a heating rate of 1 °C per min at the temperatures from 30 to 95 °C and excess pressure of 3.0 atm. The protein concentration was 1.2 mg·ml^-1^ in 20 mM potassium phosphate buffer (pH 6.5). Baseline corrections were performed and smoothed by subtracting a buffer thermogram. The data were analyzed by Launch NanoAnalyze Software (TA Instruments, USA).

Azure B decolorization was performed with 0.1 mg·ml^-1^ enzyme samples. Reaction volume contained 900 μl of 2% Azure B (Sigma, USA), 100 μl of 10 mM hydroxybenzotriazole (HOBt, Sigma, USA) and 10 μl of the enzyme. Reaction was carried out for 20 hours.

### Effects of pH and temperature on the activity and stability of the laccases

The effect of pH on the oxidation rates of various substrates was studied by measuring the enzyme activity in Britton & Robinson (B&R) buffers (pH 1.81–6.5). pH-optima for 2,2’-azino-bis(3-ethylbenzothiazoline-6-sulfonic acid) diammonium salt (ABTS, Sigma, USA), pyrocatechol (Sigma, USA), 2,6-dimethoxyphenol (2,6-DMP, Sigma, USA), gallic acid (Sigma, USA) and syringaldazine (Sigma, USA) were measured using substrate concentrations of 1 mM for ABTS, 2,6-DMP, ferulic acid, sinapic acid and gallic acid, 5 mM for guaiacol, 10 mM for pyrocatechol and 0.042 mM for syringaldazine.

Temperature optima of the laccases were determined by using pyrocatechol as a substrate in 0.1 M Na-acetate buffer, pH 4.5. Enzymatic reaction rate was determined in the range of 25–80 °C using an integrated Peltier element (PerkinElmer, USA).

Thermal stability was measured after previous incubation of the enzyme in a concentration of 0.1 mg·ml^-1^ in 50 mM potassium phosphate buffer pH 6.5 at 60 and 70 °C, and residual activity was assayed with the pyrocatechol as a substrate.

### Kinetic studies of the laccases

Kinetic constants were measured spectrophotometrically using a PerkinElmer Lambda 35 spectrophotometer (USA) at 25°C. Substrate concentrations were 0.05–10 mM for pyrocatechol, 0.005–1 mM for ABTS and 2,6-DMP, 0.005–0.15 mM for ferulic and sinapic acids, 0.05–5 mM for guaiacol, 0.05–1 mM for gallic acid, and 0.0021–0.042 mM for syringaldazine. The molar extinction coefficients were 740 M^-1^·cm^-1^ at 410 nm for catechol, 29500 M^-1^·cm^-1^ at 436 nm for ABTS, 35645 M^-1^·cm^-1^ at 470 nm for 2,6-DMP, 4610 M^-1^·cm^-1^ at 385 nm for gallic acid, 65000 M^-1^·cm^-1^ at 525 nm for syringaldazine, 14640 M^-1^·cm^-1^ at 306 nm for sinapic acid, 12940 M^-1^·cm^-1^ at 314 nm for ferulic acid, 6490 M^-1^·cm^-1^ at 464 nm. 10 μl of enzyme solution at a concentration of 1 mg·ml^-1^ (50 mM potassium phosphate buffer pH 6.5) was added to 2 ml substrate solution in 0.1 M McIlvaine buffer, pH 4.5. The reaction rate was measured by monitoring of the absorbance change during 3 min.

The kinetic parameters of pinoresinol oxidation were determined by monitoring of the oxygen consumption using an MTH-001 potentiostat (Econix-Expert, Moscow, Russia), equipped with a Clark-type electrode. The reactions were performed at 25 °C. The laccase solution (25 μl) with a concentration of 0.1 mg·ml^−1^ was added to 2000 μl of the substrate solution (10–1000 μM) in 0.1 Mcitrate-phosphate buffer pH 4.5.

Kinetic constants were calculated by non-linear fitting using the Origin-Lab program (Northampton, MA, USA). All measurements were performed at least in triplicate.

Protein concentration was measured with the BCA Protein assay kit (Pierce, USA) according to the manufacturer’s instructions.

## Results

### Strains verification and phylogenetic positioning

Fungal isolates LE-BIN 2013 and LE-BIN 2738 were identified as *Junghuhnia nitida* and *Steccherinum bourdotii* respectively based on morphological examination of pure cultures in laboratory conditions. The strains demonstrated somewhat similar micromorphological characters. Long generative hypha with numerous clamps on almost every septa, hyphal anastomoses, intercalary and terminal chlamidospores-like swellings, and abundant crystals were registered in mycelium of both strains (Table A, Figures B and C in S1 File). On BWA the mycelial mat of the *S*. *bourdotii* strain characterized by fibrous radially ordered hyphae with denser yellowish cords in the central part of the colony. Purple pigments and early primordia could be seen in inoculum zone (Fig 1A, panel 2). The colony of the *J*. *nitida* strain had thin hyphae with yellowish fluffy zones of secondary mycelium (Fig 1A, panel 1). The strains differed in growth rates and fruiting-bodies morphology (Fig 1A and 1B). The mean growth rate on BWA plates of *J*. *nitida* (6.7 mm/day) was almost twice higher compared to *S*. *bourdotii* (4.0 mm/day). Colony radii on the first week were 34.6 ± 0.5 mm for *J*. *nitida* and 8.4 ± 0.2 mm for *S*. *bourdotii*. Plate surface was entirely covered by fungal mycelium in two weeks in the case of *J*. *nitida* and in four weeks in the case of *S*. *bourdotii*. The strains of both species easily fruited in the culture on sawdust substrate with formation of typical for *Junhuhnia* genera dentate polypore hymenophore and typical for *Steccherinum* genera hydnoid one (Fig 1B, panel 1). Cultured basidiomata of the *S*. *bourdotii* strain had characteristic purple pigmentation (Fig 1B, panel 2).

Phylogenetic analysis of ITS1, ITS2 and 5.8S rDNA sequences confirmed morphological identification of studied fungi and their distinctiveness from typical core polyporoid clade fungi (Fig 2). Both *S*. *bourdotii* LE-BIN 2738 and *J*. *nitida* LE-BIN 2013 sequences form well supported clusters (100 bootstraps value) with sequences from other fungi of the same genera previously deposited in the GeneBank. Fungi of *Junghuhnia* and *Steccherinum* genus form two distinct clades inside *Steccherinaceae* group, which is distinct from the fungi clade containing *Trametes* and *Coriolopsis* genus. Ribosomal gene sequences of fungi from *Steccherinaceae* group of the residual polyporoid clade have on average 85% pair-wise identity, whereas average pair-wise identity between ribosomal gene sequences of fungi from residual polyporoid clade (*Steccherinaceae* group) and core polyporoid clade (*Trametaceae* group) in is about 70%.

### Oxidative potential of *J nitida* and *S*. *bourdotii* strains

Weekly evaluation of overall oxidoreductase and lignin peroxidase activities showed that both *J*. *nitida* and *S*. *bourdotii* have high oxidative potential (Table 1).

**Table 1:** Oxidative potential of *Junghuhnia nitida* and *Steccherinum bourdotii* strains evaluated by the express method.

Strain	Cultivation time, weeks	Activity		
		syringaldazine	guaiacol	Azure B
LE-BIN 2013 *Junghuhnia nitida*	1	+	+	-
	2	++	+++	+
	3	+++	+++	++
	4	+++	+++	+++
LE-BIN 2738 *Steccherinum bourdotii*	1	nd	nd	-
	2	+++	+++	-
	3	+++	+++	++
	4	++	+++	+++

The activity was evaluated visually by the color reaction intensity on a scale from “–" (no activity) to"+++" (the highest activity); nd–non detectable (see in text)

In the case of the overall oxidoreductase activity, for the *J*. *nitida* strain maximal activity toward guaiacol and syringaldazine was detected at the second and the third week of growth, respectively, and at the fourth week the activity remained high. For the *S*. *bourdotii* strain due to the slower colony growth the evaluation of the overall oxidoreductase activity at the first week of cultivation was impossible. At the second and the third weeks of cultivation the activity toward both guaiacol and syringaldazine became maximal. At the fourth week of cultivation the activity toward syringaldazine decreased, while activity toward guaiacol remained at the high level.

In the case of the lignin peroxidase, *J*. *nitida* activity increased more rapidly relative to *S*. *bourdotii*. *J*. *nitida* fully decolorized Azure B at the third week (Fig 1C, panel 1), while *S*. *bourdotii* fully decolorized Azure B at the fourth week (Fig 1C, panel 2). The slower Azure B decolorization by *S*. *bourdotii* could be attributed to the slower growth of this fungus. For both strains the test plates were slightly darker than the control plates with Azure B decolorized by *T*. *hirsuta*. This could result from the pigmentation of the medium by *J*. *nitida* and *S*. *bourdotii*.

**Fig 3:**
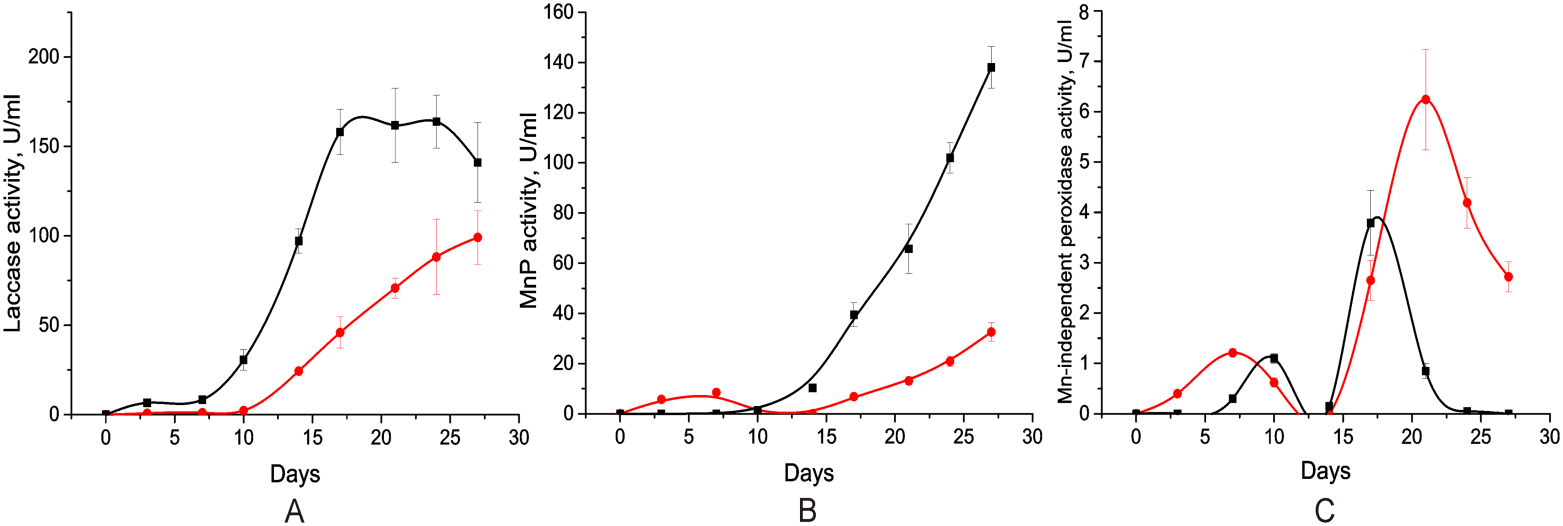
The activity profiles of the lignin degrading enzymes during *J*. *nitida* (red circles) and *S*. *bourdotii* (black squares) static surface cultivation on LC medium. Panels A-C show the activities of laccase, manganese peroxidase and Mn^2+^- independent peroxidases (versatile peroxidase and/or lignin peroxidase), respectively.

To asses ligninolytic enzyme production in the conditions that are closer to the natural fungal environment, the *J*. *nitida* and *S*. *bourdotii* strains were grown on GP medium supplemented with lignocellulose (LC medium). The dynamic of the activities of lignin degrading enzymes such as laccase and peroxidases (MnP, LiP and/or VP) was periodically measured (Fig 3).

The profiles of laccase activity for both fungi had similar sigmoidal shapes. The initial lag period for *J*. *nitida* and *S*. *bourdotii* was 10 and 7 days, respectively. In the case of *J*. *nitida*, the laccase activity started to reach plateau phase at the end of the cultivation. For *S*. *bourdotii* it took 15 days to reach the plateau, and the laccase activity maintained at the same level up to the 25^th^ day (Fig 3A).

The profile of MnP activity of *J*. *nitida* had a weak peak on the 7^th^ day of growth, and then increased steadily. In contrast, MnP activity of *S*. *bourdotii* was growing with lag period of 7 days during all cultivation (Fig 3B).

The activity profiles of Mn^2+^- independent peroxidases for both fungi were bimodal with the weaker first peaks. For *J*. *nitida* The LiP and/or VP activity peaks occurred on the 7^th^ and the 21^st^ day, while for *S*. *bourdotii* the peaks were closer in time and occurred on the 10^th^ and the 17^th^ days of growth (Fig 3C).

It should be noted, that for both fungi significant laccase production occurred earlier than production of peroxidases. The overall laccase and MnP production for both fungi was much higher than the production of Mn^2+^- independent peroxidases.

### Laccase production, purification and identification

In order to study the laccase production by *J*. *nitida* and *S*. *bourdotii* the strains were cultivated for 25 days by submerged method in the liquid medium supplied with CuSO_4_ as an inducer of laccase biosynthesis. The maximal peaks of laccase activity for both fungal strains (4–6 U·ml^-1^ for *J*. *nitida* and 8–10 U·ml^-1^ for *S*. *bourdotii*) were observed at the 20-22^th^ cultivation days after which activity remained at the same level up to 25^th^ day (Figure D in S1 File).

Monomeric laccases, SbL and JnL, were purified by 47 and 49-fold with 35 and 20% yields from the fermentation broth of *S*. *bourdotii* and *J*. *nitida* using (NH_4_)_2_SO_4_ precipitation followed by two steps of anion exchange and one step of gel-filtration column chromatography. The specific activities for SbL and JnL were 180 and 250 U·mg^-1^ respectively.

**Fig 4:**
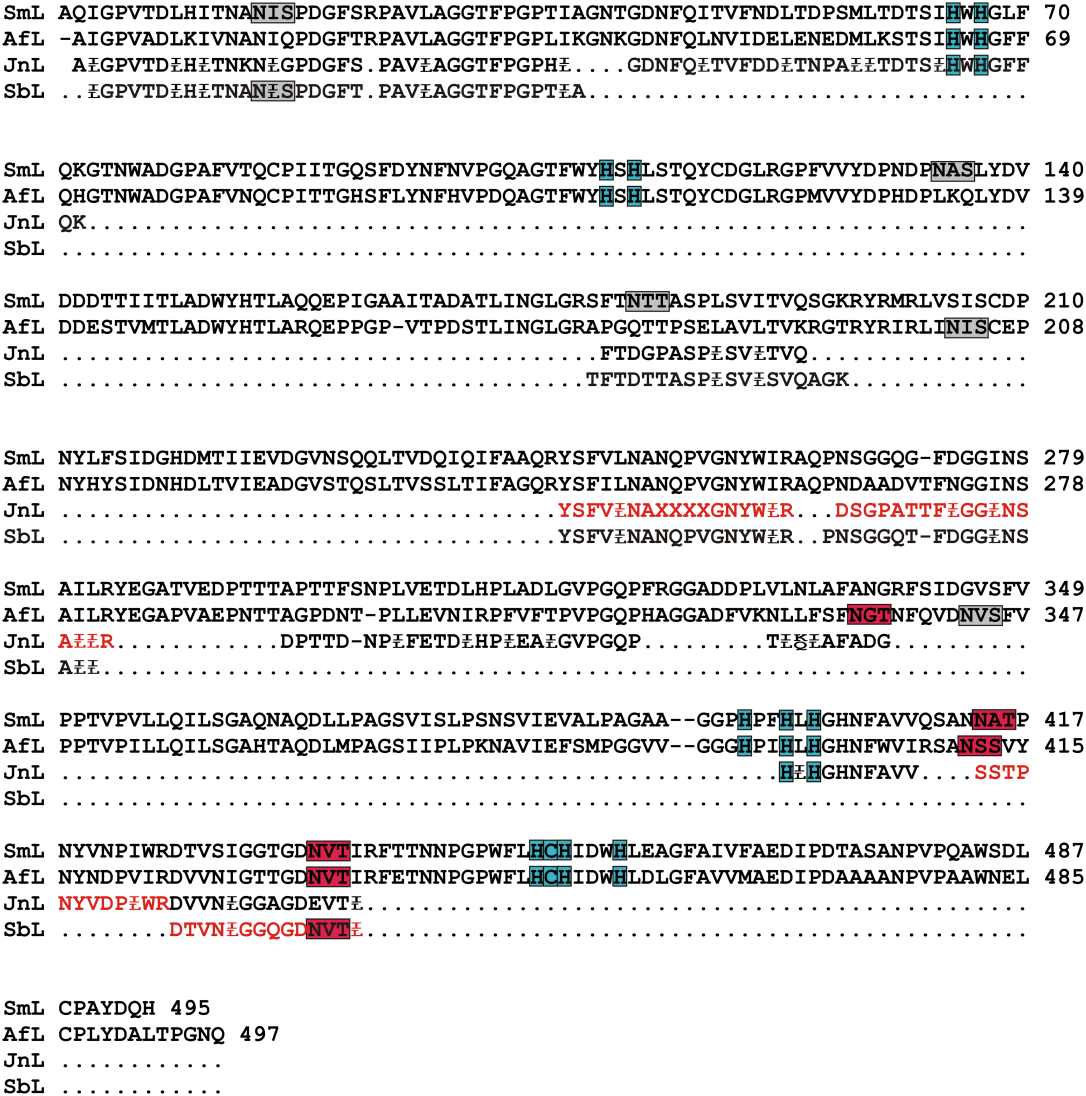
The *de novo* sequenced peptides of JnL and SbL aligned with the amino acid sequences of laccase 2 from *S*. *murashkinskyi* (SmL) and laccase A from *A*. *faginea* (AfL). The glycosylated peptides are shown in red. The potential glycosylation sites are highlighted in grey, and the occupied glycosylation sites are highlighted in red. The coordinating ligands of the copper ions are highlighted in turquoise.

SbL and JnL identification was performed by MALDI-TOF/TOF mass-spectrometry. The fragmentation spectra of the peptides with maximal intensity were obtained for both laccases (Fig 4, Table B in S1 File). Both JnL and SbL were identified as laccases based on the *de novo* obtained amino acid sequences of peptides. Almost all amino acid sequences of SbL and JnL peptides showed the highest identity to the sequences of the laccase 2 from *S*. *murashkinskyi* (AFI41889.1; PDB code 5E9N) or laccase A from *A*. *faginea* (ALE66817.1; PDB code 5EHF).

### Laccase characterization

**Fig 5:**
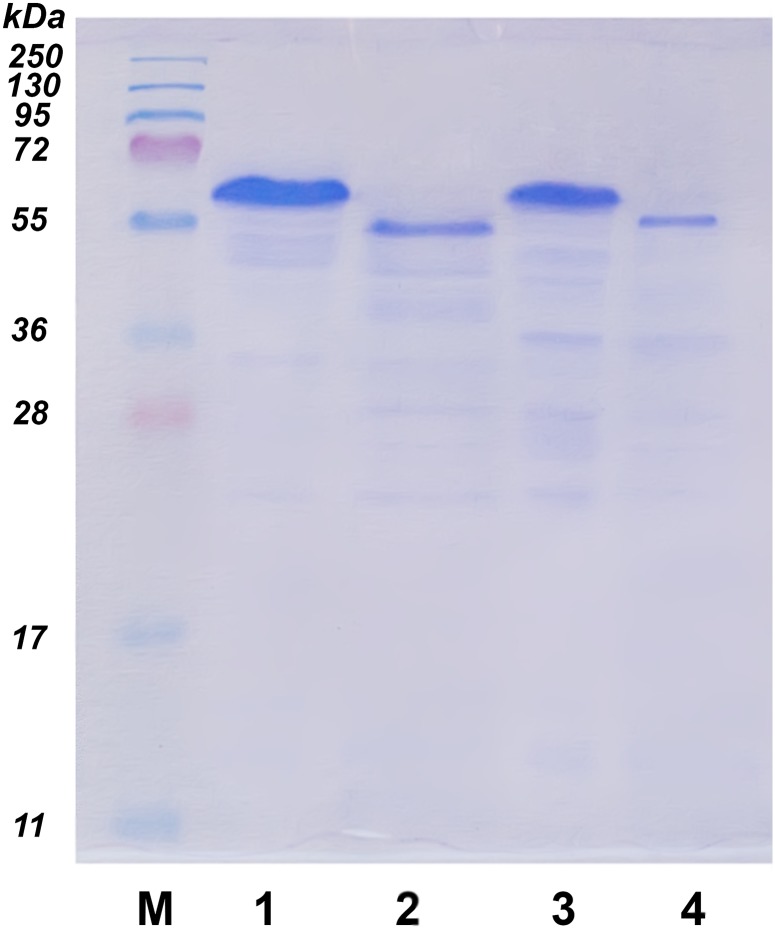
SDS-PAGE of JnL and SbL. Lines: M—molecular mass markers, 1 –JnL, 2 –JnL treated with PNGase F, 3 –SbL, 4 –SbL treated with PNGase F.

SbL and JnL preparations had the typical blue color of the multicopper oxidases. Their UV-Vis absorption spectra (Figure E in S1 File) showed a peak at 610 nm and a shoulder at 330 nm indicating the presence of type 1 and type 3 copper ions. The absorbance ratio between 280/610 nm was 23 and 21.3 for SbL and JnL, respectively. The molecular weights of SbL and JnL were approximately 63 kDa (Table 2 and Fig 5), which is typical for fungal laccases [14]. The p*I*s of SbL and JnL determined by IEF-PAGE were 3.1 and 3.4, respectively (Table 2).

**Table 2:** Physicochemical characteristics of the laccases.

Laccase	SbL	JnL	ThL	CcL
Molecular weight, kDa	63	63	66 [34]	63 [35]
p*I*	3.1	3.4	4.0 [34]	3.5 [35]
Carbohydrate content, %	8	13	12 [34]	16 [35]
*E*^o^_T1_, mV (vs. NHE)	640±20	610±20	780±10 [33]	780±10 [35]
Temperature optima, °C	70	70	55 [34]	65–70
pH-optima:				
Pyrocatechol	4.3	3.8	4.5	4.9
Gallic acid	4.0	4.0	4.0	4.1
2,6-DMP	3.8	3.7	3.5	3.5
Sinapic acid	4.0	3.9	3.8	4.4
Syringaldazine	4.1	4.5	4.5	4.7
Guaiacol	3.9	4.3	3.8	4.8
Ferulic acid	4.4	4.6	4.2	4.6
ABTS	3.0	3.0	3.0	2.5
τ_1/2_ (60 °C), min	1100	350	20	200
τ_1/2_ (70 °C), min	135	40	< 1	9.5
DSC parameters:				
Δ*H*cal, kJ/mol	1135.6	1055.8	287.0	511.6
*T*_max_, °C	85.1	81.3	69.4	77.0

The deglycosylation of the laccases with PNGase F resulted in a decrease of the molecular weight by approximately 8 kDa for SbL and 5 kDa for JnL, indicating that the carbohydrate content of SbL and JnL was about 8 and 13%, respectively.

For JnL the MALDI TOF/TOF analysis revealed carbohydrate residues in the peptides with m/z 2206, 2234, 2923, 3085, 3381 (Table B in S1 File, Fig 4). The peptides with m/z 2923, 3085 and 3381 corresponded to the same amino acid sequence SSTPNYVDP(I/L)WR. So, at least three glycosylation sites were detected for JnL. For SbL three peptides with m/z 2775, 2937 and 4496 contained carbohydrate residues. The peptides with m/z 2775 and 2937 corresponded to the same amino acid sequence DTVN(I/L)GGQGDNVT(I/L)R. Due to the low quality of the fragmentation spectra of the peptide with m/z 4496 its sequence could not be determined. So, one or two glycosylation sites were detected for SbL. In the case of the deglycosylated samples of JnL and SbL, the peaks corresponding to the glycosylated peptides disappeared and some new peptides (m/z 1910 for JnL and m/z 1761 for SbL) appeared in mass spectra. Both peptides corresponded to the same amino acid sequences as previously described peptides (Table B in S1 File) but did not contain carbohydrate residues.

**Fig 6:**
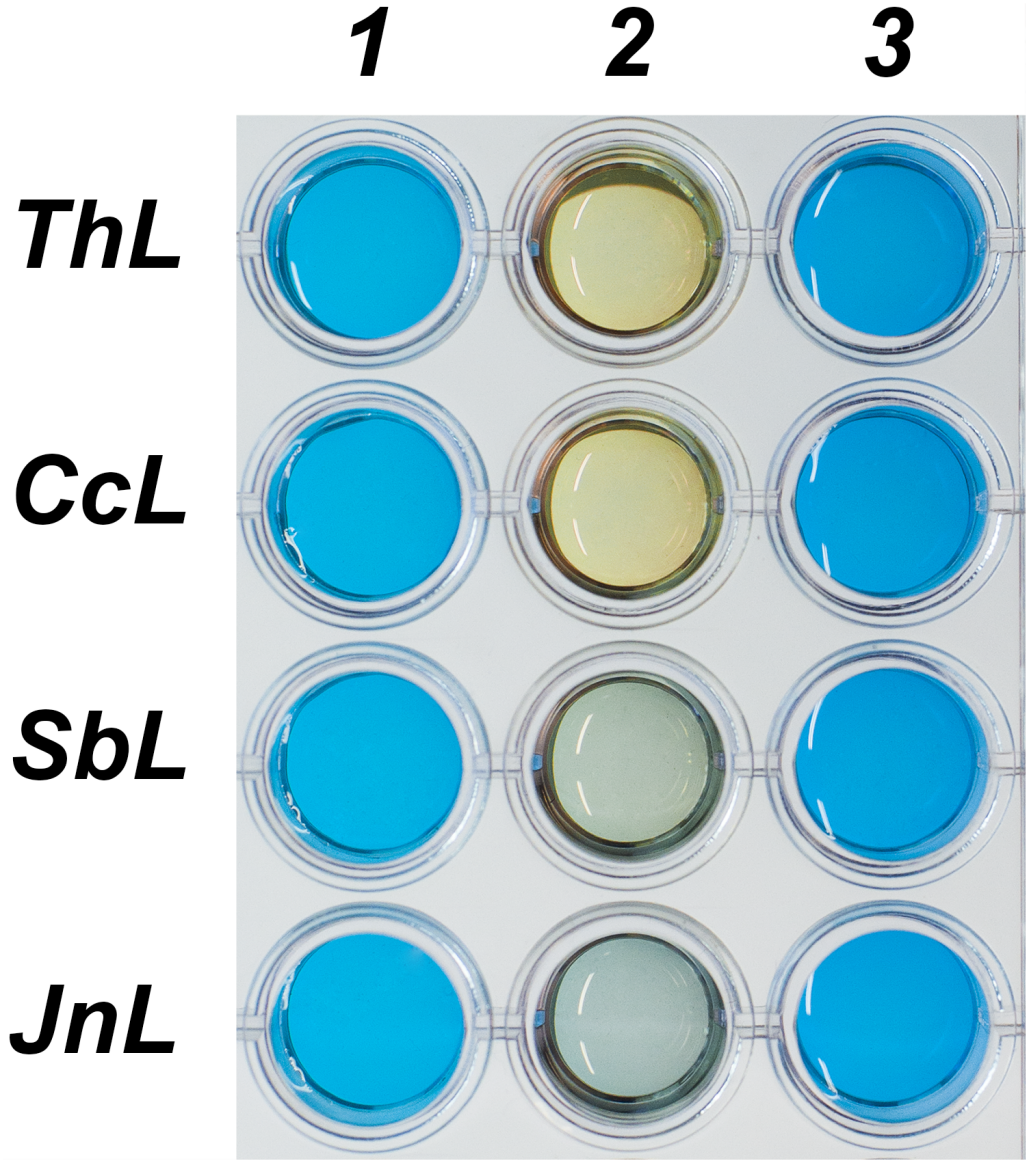
Azure B decolorization by different laccases. 1 –Azure B+HOBt, 2 –Azure B + HOBt+enzyme, 3 –Azure B.

Simple estimation of JnL and SbL T1 copper center redox potentials was performed with Azure B decolorization test. The laccases ThL and CcL with known redox potentials (780 mV for each) were used as references. The high-redox potential laccases ThL and CcL completely decolorized Azure B in 20 hours in the presence of HOBt (Fig 6). At the same time decolorization rates of Azure B by JnL and SbL were much lower. Also, JnL decolorized Azure B slower, than SbL. So, it could be assumed that these laccases possess lower redox potential than ThL and CcL.

Precise values of the T1 copper center redox potentials of JnL and SbL were measured by direct redox titration with mediators. The redox titration curves are shown in Figure F in S1 File. The redox potentials of JnL and SbL were 610 and 640 mV vs NHE, respectively. So, both JnL and SbL could be considered as middle-redox potential laccases.

Both JnL and SbL are thermostable enzymes. Their temperature optima assessed with pyrocatechol as a substrate were 70 °C (Table 2), which is quite similar to CcL and higher than ThL temperature optima. The periods of half-life for SbL and JnL at 60 °C were 1100 and 350 minutes, while at 70 °C—135 and 40 minutes, respectively (Table 2). Thus, SbL was more thermostable than JnL, and both these laccases were much more thermostable than CcL and ThL.

**Fig 7:**
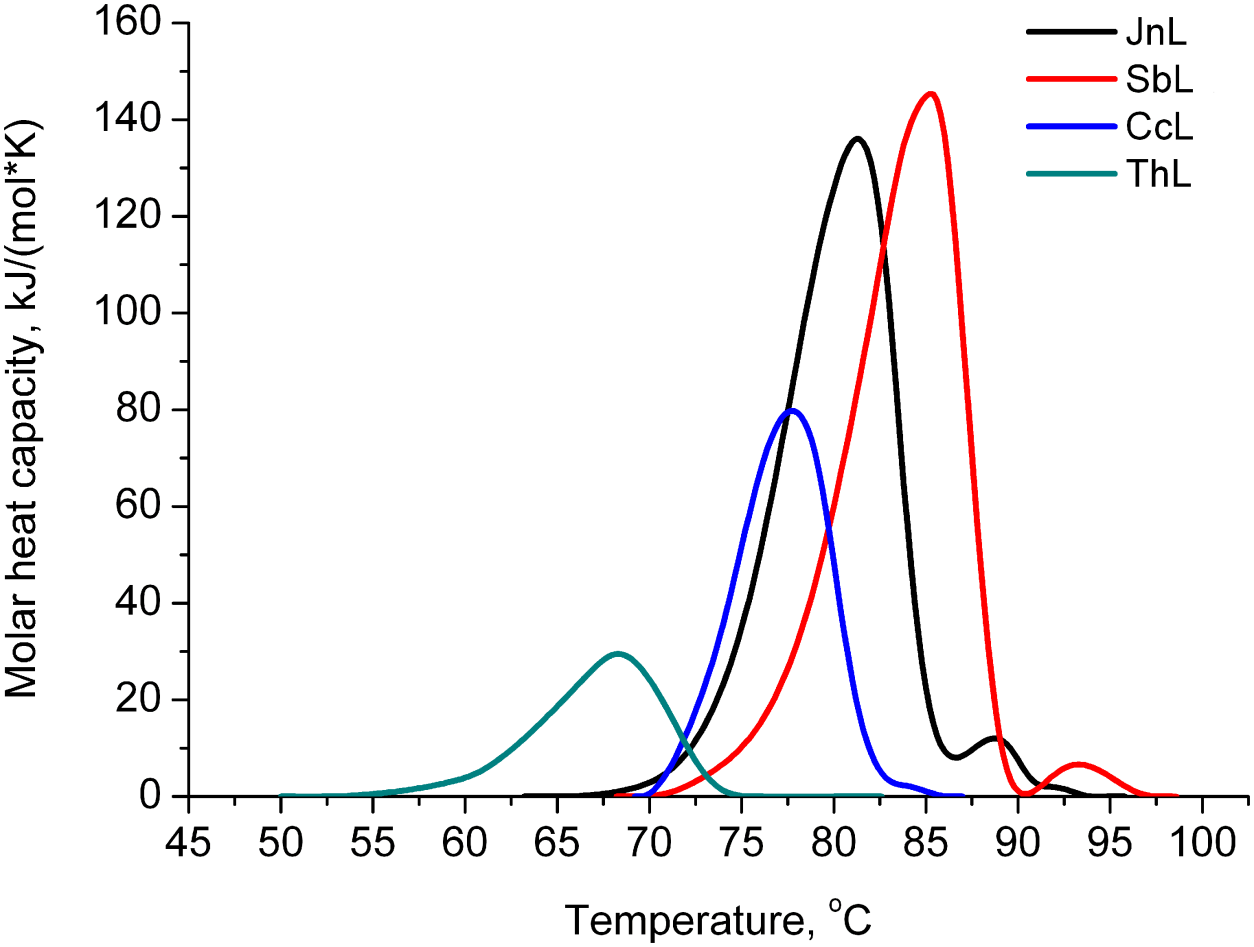
DSC melting curves of ThL, CcL, JnL and SbL laccases.

To evaluate structural stability and to estimate the melting temperature (*T*_max_), all studied laccases were taken for DSC analysis. The results showed that SbL was denatured at higher temperature (*T*_max_ of 85.1 °C) than JnL (*T*_max_ of 81.3 °C) (Fig 7). For ThL and CcL *T*_max_ were lower and reached 69.4 °C and 77.0 °C, respectively, which is in line with lower thermal stability of these laccases. However, the enthalpy of denaturation, which is associated with the proteins unfolding, for both SbL and JnL had relatively equal values such as 1135.6 and 1055.8 kJ·mol^-1^, respectively. Corresponding enthalpy values for ThL and CcL were 3-fold and 2-fold lower (Table 2). DSC profiles of SbL and JnL had an additional peak at higher temperature that allowed assuming the presence of the second calorimetric domain. Higher thermal stability of JnL and SbL could not be attributed to the enzyme carbohydrate contents, which is lower, compared to less thermostable ThL and CcL. This situation was previously observed by other researchers [36,37].

The pH-optima were determined for seven phenolic substrates and non-phenolic substrate, ABTS. For the phenolic substrates the pH optima of all four laccases were in the acidic pH range of 3.5–4.9 (Table 2). For gallic acid, 2,6-DMP and ferulic acid the pH-optima of all four laccases were almost the same. The greatest variability in the pH optima were shown for the guaiacol and pyrocatechol oxidations, with CcL having the highest pH-optima (approximately 4.8) for both of these substrates. The pH-profiles for all studied phenolic substrates had a typical bell shape and none of the laccases remained active at pH higher than 6.5 indicating the inhibitory effect of the OH^-^ ions on the laccases [38]. For non-phenolic substrate, ABTS, CcL showed the lowest pH-optima (2.5), while for the other laccases the pH-optima were identical (3.0). Obtained data are typical for fungal laccases. While pH optima for the oxidation of ABTS are generally lower than 4.0, phenolic compounds like 2,6-DMP, catechol and syringaldazine exhibit higher pH optima in the range between 4.0 and 7.0 [14].

### Catalytic properties of the laccases

Kinetic parameters for pyrocatechol, 2,6-DMP, guaiacol, gallic acid, sinapic acid, ferulic acid, syringaldazine, ABTS and pinoresinol oxidation by JnL, SbL, ThL and CcL are reported in Table 3. The kinetic study of JnL, SbL, CcL and ThL showed that all laccases exhibited similar Michaelis constants for oxidation of non-phenolic substrate ABTS. All four laccases most efficiently oxidized 2,6-DMP, sinapic acid, ferulic acid and syringaldazine. It is interesting that syringyl-type substrates like 2,6-DMP, sinapic acid and syringaldazine were better in terms of *K*_m_ and *k*_cat_/*K*_m_ values for JnL and SbL compared to CcL and ThL. Among all tested substrates maximum *k*_cat_/*K*_m_ values for JnL, SbL and CcL were observed with syringaldazine, while for ThL—with sinapic acid. In case of the model lignin compound, pinoresinol, ThL showed the highest affinity (the lowest *K*_m_) and *k*_cat_/*K*_m_ values among all studied laccases.

**Table 3:** The kinetic parameters of oxidation of the various substrates by the laccases.

Substrate		*Steccherinaceae* group		*Trametaceae* group	
		JnL	SbL	CcL	ThL
ABTS *E*^0^ = 681 mV^1^ [39]	*k*_cat_/*K*_M,_ s^-1^mM^-1^	7176	13824	11813	13235
	*K*_M,_ μM	17±2	17±2	16±2	17±2
	*k*_cat_, s^-1^	122±3	235±5	189±4	225±17
Pyrocatechol *E*^0^ = 608 mV^1^ [39]	*k*_cat_/*K*_M,_ s^-1^mM^-1^	3065	806	330	1650
	*K*_M,_ μM	62±19	222±43	815±65	183±16
	*k*_cat_, s^-1^	190±7	179±7	269±6	302±27
Gallic acid *E*^0^ = 473 mV^2^ [40]	*k*_cat_/*K*_M,_ s^-1^mM^-1^	477	417	434	260
	*K*_M,_ μM	109±16	158±22	129±18	223±32
	*k*_cat_, s^-1^	52±2	40±2	55±2	58±3
Guaiacyl-type phenolic compounds					
Guiacol *E*^0^ = 679 mV^3^ [41]	*k*_cat_/*K*_M,_ s^-1^mM^-1^	897	333	237	976
	*K*_M,_ μM	145±11	409±18	696±50	173±15
	*k*_cat_, s^-1^	130±2	136±2	165±12	162±10
Ferulic acid *E*^0^ = 692 mV^2^ [40]	*k*_cat_/*K*_M,_ s^-1^mM^-1^	6947	5579	11700	9643
	*K*_M,_ μM	19±1	19±2	20±2	28±3
	*k*_cat_, s^-1^	132±2	106±3	234±4	270±25
Pinoresinol *E*^0^ = 1670 mV^4^ [42]	*k*_cat_/*K*_M,_ s^-1^mM^-1^	651	403	578	1197
	*K*_M,_ μM	89±12	149±22	147±18	61±8
	*k*_cat_, s^-1^	58±2	60±3	85±4	73±3
Syringyl-type phenolic compounds					
2,6-DMP *E*^0^ = 580 mV^4^ [43]	*k*_cat_/*K*_M,_ s^-1^mM^-1^	12571	12375	1862	5333
	*K*_M,_ μM	7±1	8±1	65±5	24±2
	*k*_cat_, s^-1^	88±2	99±1	121±8	128±11
Sinapic acid *E*^0^ = 620 mV^2^ [40]	*k*_cat_/*K*_M,_ s^-1^mM^-1^	34000	39875	19059	24385
	*K*_M,_ μM	9±1	8±1	17±2	13±1
	*k*_cat_, s^-1^	306±15	319±17	324±31	317±23
Syringaldazine *E*^0^ = 511 mV^1^ [39]	*k*_cat_/*K*_M,_ s^-1^mM^-1^	48500	92000	24400	3853
	*K*_M,_ μM	2±0.4	1±0.3	5±0.3	34±4
	*k*_cat_, s^-1^	97±4	92±4	122±2	131±10

^1^pH = 5.0;

^2^pH = 6.0;

^3^pH = 5.6;

^4^pH = 7.0

## Discussion

The *J*. *nitida* and *S*. *bourdotii* fungi from the residual polyporoid clade were characterized and compared to the *C*. *caperata* and *T*. *hirsuta* fungi from the core polyporoid clade. The species status was confirmed by the rDNA sequencing and micromorphological study.

In nature *T*. *hirsuta*, *C*. *caperata*, *J*. *nitida* and *S*. *bourdotii* do not exhibit narrow selectivity to the host-tree and colonize predominately dead wood of angiosperms. [11,44–46]. *T*. *hirsuta* is a wound primary xylotrophic basidiomycete. It can attack living trees and contributes to the first stages of wood decomposition. *C*. *caperata* is also a primary xylotroph, performing the first stages of wood decomposition [11]. *J*. *nitida* and *S*. *bourdotii*, being secondary xylotrophs, colonize the dead wood at advanced stages of decomposition accelerating the process of its decay [47].

Express analysis of the *J*. *nitida* and *S*. *bourdotii* oxidative potentials showed that the peroxidase activity of both strains appeared only on the 2–3 week of growth. At the same time, the overall oxidoreductase activity was detected at the first week of cultivation and reached maximum values by the 2–3 week. Considering syringaldazine as a test substrate for laccase activity [11], it can be assumed that the *J*. *nitida* and *S*. *bourdotii* strains secreted laccases at first, and only then peroxidases.

During the growth of the fungi on the lignocellulose substrate, the laccase activity, in general, also precedes the peroxidase activity. Moreover, on 10-15^th^ days the overall laccase activity was significantly higher than peroxidase activity. In contrast, during cultivation of *T*. *hirsuta* on various media, secretion of peroxidases was shown to precede laccase secretion [22,48]. Such a difference in the secretion profiles of ligninolytic enzymes may be due to the fact that *J*. *nitida* and *S*. *bourdotii* belong to the group of secondary xylotrophs, and *T*. *hirsuta* belongs to the group of primary xylotrophs. In the process of lignin destruction, the primary xylotrophs accumulate, by the action of peroxidases, various toxic phenolic products. Therefore, when the wood substrate is colonized by secondary xylotrophs, these fungi have to detoxify the accumulated phenolic components, and only after this begin to synthesize peroxidases necessary for the further destruction of the lignocellulosic substrate.

Both *J*. *nitida* and *S*. *bourdotii* can be considered as an active laccase producers, since the laccase activity in their cultural broth during cultivation on the glucose peptone medium with Cu^2+^ is comparable with those in broths of some *Trametes* genus representatives [11]. However, in the case of effective laccase producers from *Trametes* genus, such as *T*. *hirsuta* and *C*. *caperata*, laccase activity reached the maximum at 12–14 days of a submerged cultivation and then dramatically falls at 16–18 days [11,34]. In contrast, the laccase activity of *J*. *nitida* and *S*. *bourdotii* increased gradually for 20–22 days and subsequently remained at the same level up to 25 days (Table 1, Fig 3).

Currently, one of the most studied laccases are those derived from the *Coriolopsis* and *Trametes* fungal groups. Although these laccases can possess very diverse properties, mostly they have low thermal stability at temperatures above 60 °C [37]. On the contrary, laccases from *Steccherinaceae* fungi are poorly studied, and the only representatives from this group with known properties, *S*. *ochraceum* laccases, have increased thermal stability with a half-life about 5–6 hours at 60 °C and about 1.7 hour at 70 °C [9]. Since both laccases from *J*. *nitida* and *S*. *bourdotii* also have long half-lives at 60 °C and 70 °C, we suppose that laccases from *Steccherinaceae* fungi can have enhanced thermal stability.

Glycosylation is suggested to have an effect on a protein stability by stabilization of protein conformation [37]. The carbohydrate content of JnL, SbL, ThL and CcL laccases is similar and ranges between 8 and 16% (Table 2). Interestingly, the SbL, which have the lowest carbohydrate content among all laccases, have the highest thermal stability. Hence, differences in thermal stability of different laccases may be not due to carbohydrate content but due to the position of the glycosylation sites. Comparison of the sequences of JnL peptides with known laccase structures showed that JnL has one glycosylation site (peptide SSTPNYVDP(I/L)WR) typical for all structurally characterized laccases of *Steccherinaceae* fungal group (i.e. *A*. *faginea*, *S*. *murashkinskyi* and *S*. *ochraceum*) [49]. Two other sites were not previously observed in the structures of other laccases. SbL has one localized glycosylation site (peptide DTVN(I/L)GGQGDNVT(I/L)R) that is conservative for all laccase with known structures, except of *Coprinus cinereus* laccase [49]. Interestingly in JnL this site is absent (Fig 4, Table B in S1 File).

Both the classical redox titration and simple Azure B decolorization methods showed that SbL and JnL have the middle redox potentials of the T1 copper center. Moreover, Azure B decolorization rate in presence of HOBt correlates with the value of the redox-potential. So, Azure B decolorization could be proposed as a test for rough estimation of the redox potential of laccases upon the availability of several reliable control laccases with known potential.

It is believed that the catalytic efficiency of different substrates oxidation is correlated with a redox potential difference (Δ*E*^0^) between a substrate and a laccase [50]. However, the catalytic efficiency depends not only on the redox-potential difference but also on the steric features like shape of the substrate and shape and structure of the substrate-binding pocket of the laccase [51–53]. In our case, despite their middle redox potential, SbL and JnL could oxidize a broad range of substrates, including monoaromatic phenols (pyrocatechol, 2,6-DMP, guaiacol, sinapic and gallic acids), complex phenol (syringaldazine), natural dilignan (pinoresinol) and the non-phenolic heterocyclic compound ABTS, like high-redox potential laccases ThL and CcL (Table 3). In many cases middle-redox potential laccases JnL and SbL were more effective than high-redox potential laccases ThL and CcL (Table 3). There was a weak correlation between Δ*E*^0^ and catalytic constants *k*_cat_ for pyrocatechol, 2,6-DMP, syringaldazine, guaiacol, ferulic acid and pinoresinol oxidation. On the contrary, no such correlation was observed for ABTS, gallic acid and sinapic acid oxidation, indicating some other factors affecting the oxidation rate. Similar comparisons with the same conclusion were carried out earlier for several low-, middle- and high-redox potential laccases. Frasconi and coworkers [39] investigated the oxidation of different phenolic and non-phenolic compounds by fungal laccases from *Melanocarpus albomyces*, *T*. *hirsuta* and *Trametes versicolor* and plant laccase from *Rhus vernicifera*. The authors concluded that the character of the substrate binding pocket, the redox potential of the T1 copper, the reaction buffer pH and the type of substrate/mediator collectively affected the overall laccases efficiencies. Similar conclusions were done by Lahtinen et al. [54] for *M*. *albomyces* and *T*. *hirsuta* laccases on the basis of oxidation rates of model lignin compounds. It is remarkable that middle-redox potential laccases SbL and JnL showed comparable to high-redox potential laccases ThL and CcL catalytic efficiency of pinoresinol oxidation, whose potential of oxidation is 1670 mV [42]. Furthermore, the catalytic efficiency of JnL (*E*^0^_T1_ = 610 mV) towards pinoresinol was a bit higher than that of CcL (*E*^0^_T1_ = 780 mV). It can be attributed to the lower *K*_M_ value for JnL than CcL. The differences in the catalytic efficiency values could be explained by the different structures of these laccases. Some recently reported molecular evolution data based on random mutagenesis leading to mutations in the region close to the substrate binding site of laccases resulted in a more efficient phenol oxidation [55].

In contrast to CcL and ThL, *K*_M_ values for 2,6-DMP, sinapic acid and syringaldazine oxidation by JnL and SbL were very low, that indicates the highest affinity of these laccases to syringyl-type substrates (Table 3). The lowest *K*_M_ for JnL and SbL were observed for syringaldazine oxidation– 2 and 1 μM respectively. Two laccase isoforms from *S*. *ochraceum*, belonging to the same family as *S*. *bourdotii* and *J*. *nitida*, also showed low *K*_M_ value– 2 μM and 1.7 μM and high catalytic efficiency for this substrate [9]. It looks like the high affinity to syringyl-type phenolic compounds is the special feature of laccases from the *Steccherinaceae* fungi, while the laccases from *Trametes* spp. commonly exhibit lower affinity to these substrates [14], which can be connected with the different ecology of these fungi.

The substrate specialization of wood decay fungi reflects the preferences of fungi to the type of wood (softwood or hardwood), certain wood species, substrate size (logs, stems or branches) and decay stage of the wood. *S*. *bourdotii* and *J*. *nitida* as well as *T*. *hirsuta* and *C*. *caperata* grow on a hardwood substrate without any affinity to the exact wood species, preferring fallen branches and wood debris. However, *T*. *hirsuta* and *C*. *caperata* are primary xylotrophs, whereas *S*. *bourdotii* and *J*. *nitida* are secondary xylotrophs. As a secondary xylotrophs *Steccherinum* and *Junghuhnia* genera occupied an intermediate position between white-rot wood and white-rot leaf litter degrading basidiomycetes. It is interesting that the same tendency of the *S*. *bourdotii* and *J*. *nitida* laccases to show higher affinity to the syringyl-type compounds is demonstrated by some laccases from ascomycetes [15,56,57] and leaf litter decomposing basidiomycetes [58,59]. Hence, we can hypothesize that preferences of *T*. *hirsuta*, *C*. *caperata*, *S*. *bourdotii* and *J*. *nitida* to the wood at different decay stages can affect the substrate specificity of their laccases.

## Conclusion

Two new effective laccase producing species *Steccherinum bourdotii* and *Junghuhnia nitida* are found and their oxidative potentials are characterized. Both fungi demonstrated similar activity profiles of oxidative enzymes during the solid state cultivation. The laccase activity was already detectable at the early cultivation stages, while peroxidase activity was detected at the later stages. During the semisolid cultivation on the lignocellulose substrate the same tendency was observed. That can reflect the natural necessity of secondary xylotrops fungi to detoxify small phenolic lignin metabolites produced by primary xylotrops.

The laccase enzymes from these fungi were purified and characterized. Both JnL and SbL laccases had enhanced thermal stability compared with laccases from *Trametes* groups and comparable catalytic efficiency towards the different phenolic substrates, wherein its catalytic efficiency towards syringyl-type phenolic compounds was significantly higher. These observations may reflect different wood colonization strategy of *S*. *bourdotii* and *J*. *nitida*.

## Supporting information

S1 File **(Table A) Macro- and micromorphological characteristics of *Junghuhnia nitida* LE-BIN 2013 and *Steccherinum bourdotii* LE-BIN 2738 strains. (Table B) Mass-spectrometric *de novo* sequencing of *Junghuhnia nitida* LE-BIN 2013 and *Steccherinum bourdotii* LE-BIN 2738 laccases. (Figure A) Maximum likelihood (ML) phylogenetic tree of ribosomal gene sequences. (Figure B) Light microscopy examination of the *Junghuhnia nitida* LE-BIN 2013 strain**. A–generative hypha with clamps, skeletal hypha (on insertion); B–anastomoses; C–chlamidospores-like swellings; D–crystals incrustation on hypha. Bars– 10 μm. **(Figure C) Light microscopy examination of the *Steccherinum bourdotii* LE-BIN 2738 strain**. A–generative hypha with regular clamps; B–terminal and intercalary chlamidospores. Bars– 10 μm. **(Figure D) Laccase activity profiles during *J*. *nitida* (red circles) and *S*. *bourdotii* (black squares) submerged cultivation using glucose-peptone medium with CuSO**_**4**_ **as an inducer. (Figure E) UV-Vis spectra of JnL. (Figure F) Redox titrations of JnL (A) and SbL (B)**.
(DOCX)
