# Supplementary material for: White-rot basidiomycetes Junghuhnia nitida and Steccherinum bourdotii: Oxidative potential and laccase properties in comparison with Trametes hirsuta and Coriolopsis caperata
Source: PLoS One. 2018 Jun 1;13(6):e0197667. doi: 10.1371/journal.pone.0197667 (PMC5983490; doi:10.1371/journal.pone.0197667)
Supplement: S1 File — (Table A) Macro- and micromorphological characteristics of Junghuhnia nitida LE-BIN 2013 and Steccherinum bourdotii LE-BIN 2738 strains. (Table B) Mass-spectrometric de novo sequencing of Junghuhnia nitida LE-BIN 2013 and Steccherinum bourdotii LE-BIN 2738 laccases. (Figure A) Maximum likelihood (ML) phylogenetic tree of ribosomal gene sequences. (Figure B) Light microscopy examination of the Junghuhnia nitida LE-BIN 2013 strain. A–generative hypha with clamps, skeletal hypha (on insertion); B–anastomoses; C–chlamidospores-like swellings; D–crystals incrustation on hypha. Bars– 10 μm. (Figure C) Light microscopy examination of the Steccherinum bourdotii LE-BIN 2738 strain. A–generative hypha with regular clamps; B–terminal and intercalary chlamidospores. Bars– 10 μm. (Figure D) Laccase activity profiles during J. nitida (red circles) and S. bourdotii (black squares) submerged cultivation using glucose-peptone medium with CuSO4 as an inducer. (Figure E) UV-Vis spectra of JnL. (Figure F) Redox titrations of JnL (A) and SbL (B). (DOCX) [file pone.0197667.s001.docx]

**S1 File**

**Supporting Tables and Figures**

**Table A. Macro- and micromorphological characteristics of *Junghuhnia nitida* LE-BIN 2013 and *Steccherinum bourdotii* LE-BIN 2738 strains.**

|  |  | *J. nitida* LE-BIN 2013 | *S. bourdotii* LE-BIN 2738 |
| --- | --- | --- | --- |
| Macromorphology: | |  |  |
|  | *advancing zone* | submerged and appressed | submerged, appressed and raised |
|  | *margin outline* | evenly fringed | evenly fringed |
|  | *aerial mycelium* | cottony or woolly (slightly plumose when young) | woolly, plumose |
|  | *aerial mycelium hyphae* | long, radiating from inoculum with mycelial tufts | long radiating from inoculum , cords forming |
|  | *young colony* | white and farinaceous | white and farinaceous |
|  | *secondary mycelium* | yellowish and fluffy | creamy with purple pigmentation and yellowish areas |
|  | *odor* | unpleasant | unpleasant |
|  | *reversum* | remained unchanged | darkened with purple color |
| Micromorphology: | |  |  |
|  | *hyphal system* | dimitic | monomitic |
|  | *generative hypha* | long, branched, 1.3-3.5 µм in diameter with clamps almost on each septa, hyphal anastomoses, incrustations and abundant crystals presented | long, branched, 2.0-4.5 µм in diameter with clamps almost on each septa, hyphal anastomoses, rings, incrustations and abundant crystals presented |
|  | *chlamidospores* | swellings resembling intercalary chlamidospores were observed | chlamidospores 5.5-8.5 x 8.5-10.2 µм in diameter were observed |
|  | *skeletal hypha* | long, sometime branched, 2.5-3.0 µм in diameter | not observed |
| Fruiting: | |  |  |
|  | | first fruit-bodies appeared after 4 weeks of light exposition | first fruit-bodies appeared after 2 weeks of light exposition |

*Steccherinum* genus is typified by species with an effused-reflexed basidiomata and a hydnoid hymenophore, whereas for *Junghuhnia* species a dentate poroid hymenophore is typical [Miettinen O, Larsson E, Sjökvist E, Larsson K-H. Comprehensive taxon sampling reveals unaccounted diversity and morphological plasticity in a group of dimitic polypores (Polyporales, Basidiomycota). Cladistics. 2012;28: 251–270. doi:10.1111/j.1096-0031.2011.00380.x]. Cultured fruiting bodies in our experiment did not form typical basidiomata like in nature, but retained the main features characteristic of each species: *J. nitida* – dentate poroid hymenophore and *S. bourdotii –* hydnoid hymenophore.

The main difference of cultural micromorphology of *J. nitida* and *S. bourdotii* was monomitic hyphal structure of *S. bourdotii* strain – skeletal hyphae were not observed in studied microscopy slides (S2 Fig). Observation of skeletal hyphae in mycelium of *J. nitida* confirmed its dimitic hyphal structure (S1 Fig).

In our study fungi from residual polyporoid clade, *J. nitida* and *S. bourdotii,* were characterized by relatively slow growth, compared to the fungi from the core polyporoid clade, *C. caperata* and *T. hirsuta* [Fedorova T V., Shakhova N V., Klein OI, Glazunova OA, Maloshenok LG, Kulikova NA, et al. Comparative analysis of the ligninolytic potential of basidiomycetes belonging to different taxonomic and ecological groups. Appl Biochem Microbiol. 2013;49: 570–580. doi:10.1134/S0003683813060082]. Colony mats were also different – strains from the core clade usually had thick dense cotton uniform aerial mycelium getting very tight, solid and somewhat rubbery with age [Fedorova et al. 2013], whereas colonies of residual clade strains were much slender with poor spreading, often filamentous, forming radial cords, fragile aerial mycelium. In our study skeletal hypha were not observed in *S. bourdotii* culture and the hyphal system was defined as monomitic, while natural morphology of the species thoroughly described by Niemelä [Niemela T. Steccherinum bourdotii in North Europe. Folia Cryptogam Est. 1998;33: 93–97] determined it as dimitic.

**Table B. Mass-spectrometric *de novo* sequencing of *Junghuhnia nitida* LE-BIN 2013 and *Steccherinum bourdotii* LE-BIN 2738 laccases**

| m/z | Corresponding peptide sequence | BLASTp search results | | | | |
| --- | --- | --- | --- | --- | --- | --- |
|  |  | Closest homologues | Source | Accession | Score | E-value |
| *J. nitida* laccase (JnL) | | | | | | |
| 1090 | T(I/L)(K/Q)(I/L)AFADG | VLNLAFANG | laccase 2 [Steccherinum murashkinskyi] | AFI41889.1 | 20.2 | 0.012 |
| 1286 | H(I/L)HGHNFAVV | HLHGHNFAVV | laccase 2[Steccherinum murashkinskyi] | AFI41889.1 | 35.8 | 0.13 |
|  |  | HLHGHNFAVV | laccase B [Antrodiella faginea] | ALE66818.1 | 35.8 | 0.13 |
|  |  | HLHGHNFAVV | laccase [Mycena chlorophos] | GAT47314.1 | 35.8 | 0.13 |
|  |  | HLHGHNFAVV | laccase [Rhizopogonvinicolor AM-OR11-026] | OAX34405.1 | 35.8 | 0.13 |
| 1514 | DVVN(I/L)GGAGDEVT(I/L) | DVVDIGGAGDNVTI | laccase 5 [Coprinopsis cinerea okayama7#130] | XP_001829810.2 | 38.8 | 1e-02 |
|  |  | DVVSIGGAGDNVTI | laccase [Meripilus giganteus] | CBV46340.1 | 38.0 | 1e-04 |
|  |  | DVVSIGGAGDNVTI | laccase [Pholiotanameko] | ABR24264.1 | 38.0 | 1e-04 |
|  |  | DVVSIGGAGDNVTI | laccase [Laccaria bicolor S238N-H82] | XP_001886681.1 | 38.0 | 1e-04 |
|  |  | DTVSIGGTGDNVTI | laccase 2 [Steccherinum murashkinskyi] | AFI41889.1 | 38.0 | 0.004 |
| 1680 | PAV(I/L)AGGTFPGPH(I/L) | PAVLAGGTFPGPLI | laccase 3 [Steccherinum murashkinskyi] | AFI41890.1 | 39.7 | 0.001 |
|  |  | PAVLAGGTFPGPLI | laccase 9 precursor  [Cerrena sp. HYB07] | AID59414.1 | 39.7 | 0.001 |
|  |  | PAVLAGGTFPGPLI | laccase [Pisolithus microcarpus 441] | KIK22221.1 | 39.7 | 0.001 |
|  |  | PAVLAGGTFPGPLI | laccase [Punctularia strigosozonata HHB-11173 SS5] | XP_007385834.1 | 39.7 | 0.001 |
|  |  | PAVLAGGTFPGPLI | laccase [Cerrena sp. CTL-2011] | AEL16568.1 | 39.7 | 0.001 |
|  |  | PAVLAGGTFPGPLI | laccase A [Antrodiella faginea] | ALE66817.1 | 39.7 | 0.001 |
| 1817 | FTDGPASP(I/L)SV(I/L)TVQ | FTNTTASPLSVITVQ | laccase 2 [Steccherinum murashkinskyi | AFI41889.1 | 36.3 | 0.017 |
|  |  | SPQGPATPLSVITVQ | laccase [Phlebiachrysocreas] | ALF95043.1 | 36.3 | 0.017 |
| 1910 | SSTPNYVDP(I/L)WR* | NSTPNYIDPIWR | laccase B [Antrodiella faginea] | ALE66818.1 | 39.7 | 0.011 |
|  |  | NATPNYVNPIWR | Chain A, Steccherinum murashkinskyi laccase | 5E9N_A | 36.7 | 0.12 |
| 2206 | YSFV(I/L)NA(604Da)GNYW(I/L)R | YSFVLNANQPVGNYWIR | Chain A, Steccherinum murashkinskyi laccase | 5E9N_A | 42.6 | 0.001 |
|  |  | YSFVLNANQPVGNYWIR | laccase B [Antrodiella faginea] | ALE66818.1 | 42.6 | 0.001 |
|  |  | YSFVLNATQPVGNYWIR | laccase [Gelatoporia subvermispora B] | EMD32460.1 | 42.6 | 0.001 |
|  |  | YSFVLNATQPVGNYWIR | laccase precursor [Obba rivulosa] | OCH92420.1 | 42.6 | 0.001 |
| 2234 | DSGPATTF(I/L)GG(I/L)NSA(I/L)(I/L)R | PNIGNTTFLGGLNSAILR | laccase 4 precursor [Cerrena sp. HYB07] | AID59412.1 | 38.8 | 0.063 |
|  |  | NDAADVTFNGGINSAILR | Chain A, laccase from Antrodiella faginea | 5EHF_A | 33.7 | 1.7 |
| 2400 | A(I/L)GPVTD(I/L)H(I/L)TNKN(I/L)GPDGFS | QIGPVTDLHITNANISPDGFS | laccase 2 [Steccherinum murashkinskyi | AFI41889.1 | 57.1 | 2e-09 |
| 2923 | SSTPNYVDP(I/L)WR |  |  |  |  |  |
| 3085 | SSTPNYVDP(I/L)WR |  |  |  |  |  |
| 3381 | SSTPNYVDP(I/L)WR |  |  |  |  |  |
| 3577 | GDNFQ(I/L)TVFDD(I/L)TNPA(I/L)(I/L)TDTS(I/L)HWHGFFQK* | GDNFQITVFNDLTDPSMLTDTSIHWHGLFQK | Chain A, Steccherinum murashkinskyi laccase | 5E9N_A | 60.5 | 1e-09 |
|  |  | GDNFQLTVHDDLTDASMLTGTSIHWHGFFQK | laccase B [Antrodiella faginea] | ALE66818.1 | 54.3 | 1e-07 |
| 4948 | DPTTDNP(I/L)FETD(I/L)HP(I/L)EA(I/L)GVPGQP | DPTTTAPTTFSNPLVETDLHPLADLGVPGQP | Chain A, Steccherinum murashkinskyi | 5E9N_A | 56.2 | 1e-07 |
| *S. bourdotii* laccase (SbL) | | | | | | |
| 1425 | PAV(I/L)AGGTFPGPT(I/L)A | PAVLAGGTFPGPTIA | laccase 2 [Steccherinum murashkinskyi] | AFI41889.1 | 47.7 | 2e-06 |
| 1761 | DTVN(I/L)GGQGDNVT(I/L)R* | DTVNIGGPGDNVTI | Laccase-2 [Grifola frondosa] | OBZ71590.1 | 41.8 | 0.003 |
|  |  | DTVSIGGPGDNVTI | laccase [Gelatoporia subvermispora B] | EMD37153.1 | 38.4 | 0.044 |
|  |  | DTVSIGGPGDNVTI | laccase 2 [Obba rivulosa] | OCH87152.1 | 38.4 | 0.044 |
|  |  | DTVSIGGTGDNVTI | Chain A, Steccherinum murashkinskyi laccase | 5E9N_A | 37.5 | 0.088 |
| 1923 | TFTDTTASP(I/L)SV(I/L)SVQ | SFTNTTASPLSVITVQ | Chain A, Steccherinum murashkinskyi laccase | 5E9N_A | 43.5 | 0.001 |
| 1988 | PNSGGQTFDGG(I/L)NSA(I/L)(I/L) | PNSGGQTFDGGINSAIL | [laccase 2 [Steccherinum murashkinskyi]](https://blast.ncbi.nlm.nih.gov/Blast.cgi#alnHdr_385141759) | AFI41889.1 | 49.0 | 8e-07 |
|  |  | PNSGNQTFDGGINSAIL | multicopper oxidase [Hypholoma sublateritium FD-334 SS-4] | KJA22755.1 | 50.7 | 2e-07 |
| 2041 | YSFV(I/L)NANQPVGNYW(I/L)R | YSFVLNANQPVGNYWIR | [laccase 2 [Steccherinum murashkinskyi]](https://blast.ncbi.nlm.nih.gov/Blast.cgi#alnHdr_385141759) | AFI41889.1 | 61.3 | 4e-11 |
|  |  | YSFVLNANQPVGNYWIR | laccase B [Antrodiella faginea] | ALE66818.1 | 61.3 | 4e-11 |
|  |  | YSFVLNANQPVGNYWIR | laccase [Moniliophthora roreri MCA 2997] | XP_007854998.1 | 61.3 | 4e-11 |
|  |  | YSFVLNANQPVGNYWIR | laccase [Moniliophthora roreri MCA 2997] | XP_007854997.1 | 61.3 | 4e-11 |
| 2079 | TDTTASP(I/L)SV(I/L)SVQAGK* | TNTTASPLSVITVQSGK | Chain A, Steccherinum murashkinskyi laccase | 5E9N_A | 45.2 | 3e-04 |
| 2465 | (I/L)GPVTD(I/L)H(I/L)TNAN(I/L)SPDGFT | IGPVTDLHITNANISPDGFS | [laccase 2 [Steccherinum murashkinskyi]](https://blast.ncbi.nlm.nih.gov/Blast.cgi#alnHdr_385141759) | AFI41889.1 | 63.4 | 2e-10 |
| 2775 | VT(I/L)R |  |  |  |  |  |
| 2937 | VT(I/L)R |  |  |  |  |  |
| 3872 | TD(I/L)H(I/L)TNAN(I/L)SPDGFTRPAV(I/L)AGGTFPG | TDLHITNANISPDGFSRPAVLAGGTFPG | [laccase 2 [Steccherinum murashkinskyi]](https://blast.ncbi.nlm.nih.gov/Blast.cgi#alnHdr_385141759) | AFI41889.1 | 86.7 | 4e-18 |

***new peptides from mass-spectra of the deglycosylated samples**

**Peptides with carbohydrate residues detected in mass-spectra are highlighted with blue. Possible glycosylation sites are highlighted with yellow.**

**Figure A. Maximum likelihood (ML) phylogenetic tree of ribosomal gene sequences.**

**
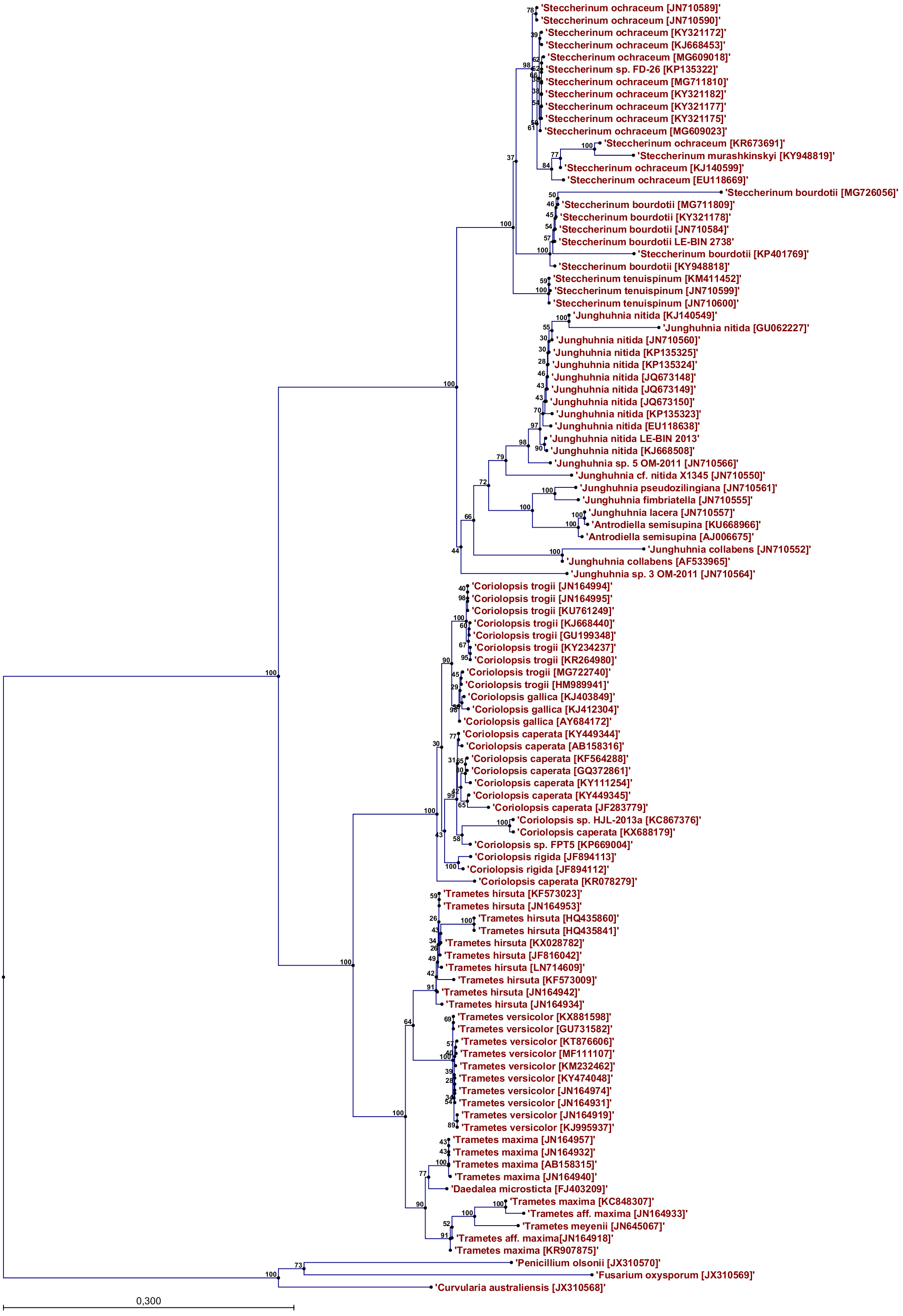
**

**Figure B. Light microscopy examination of the *Junghuhnia nitida* LE-BIN 2013 strain.** A – generative hypha with clamps, skeletal hypha (on insertion); B – anastomoses; C – chlamidospores-like swellings; D – crystals incrustation on hypha. Bars – 10 µm.


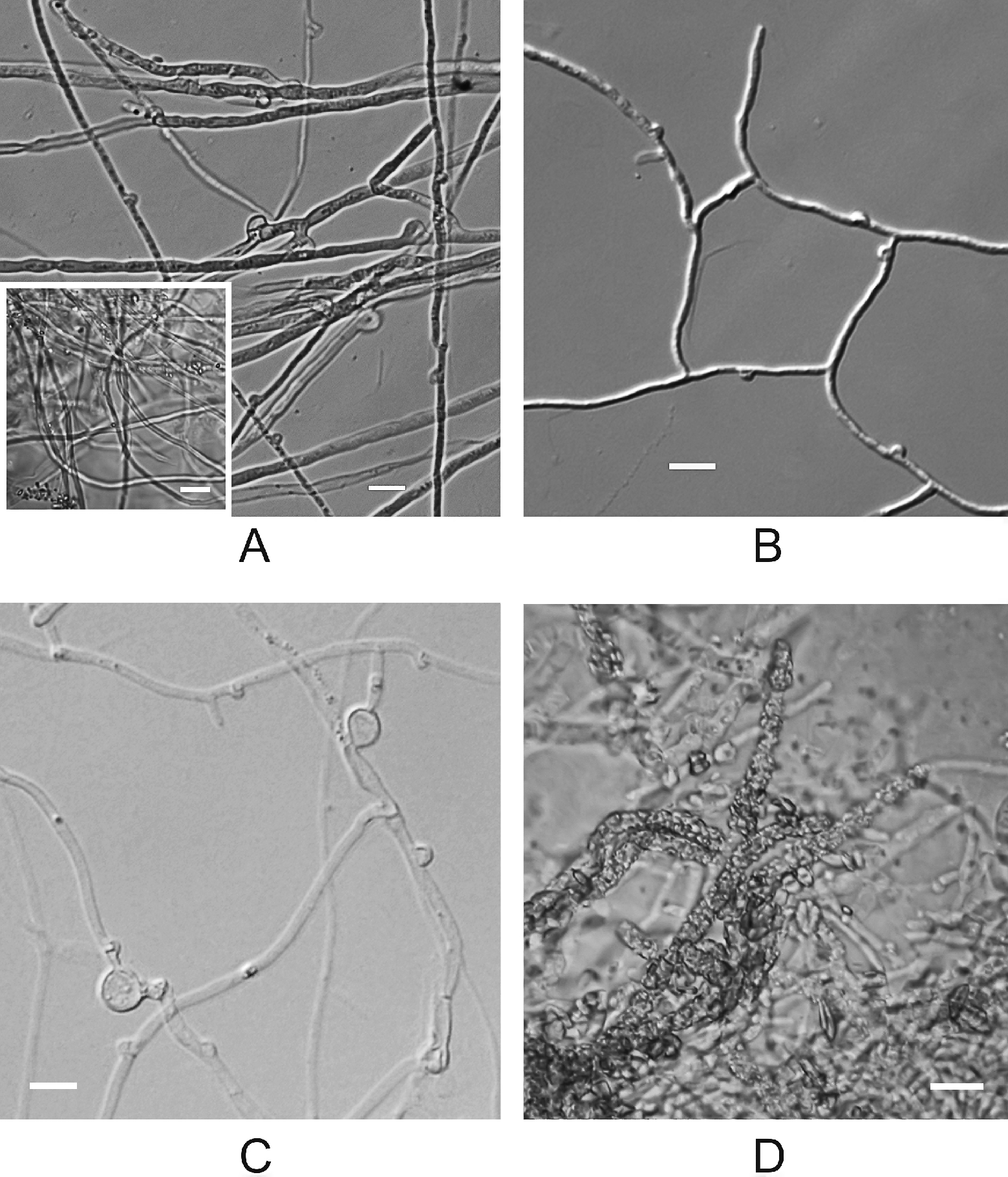


**Figure C. Light microscopy examination of the *Steccherinum bourdotii* LE-BIN 2738 strain.** A – generative hypha with regular clamps; B – terminal and intercalary chlamidospores. Bars – 10 µm.


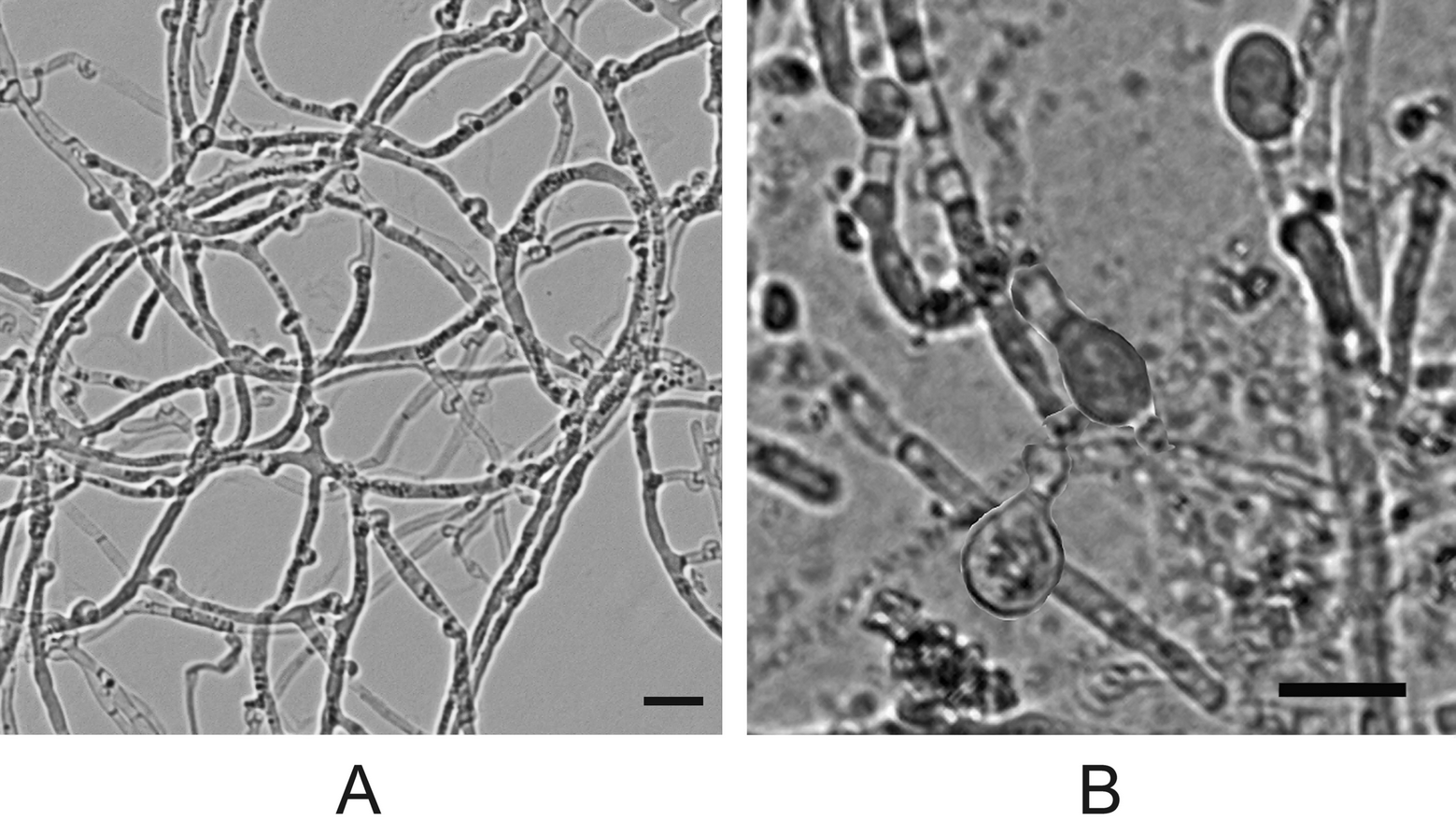


**Figure D. Laccase activity profiles during *J. nitida* (red circles) and *S. bourdotii* (black squares) submerged cultivation using glucose-peptone medium with CuSO_4_ as an inducer.**

**
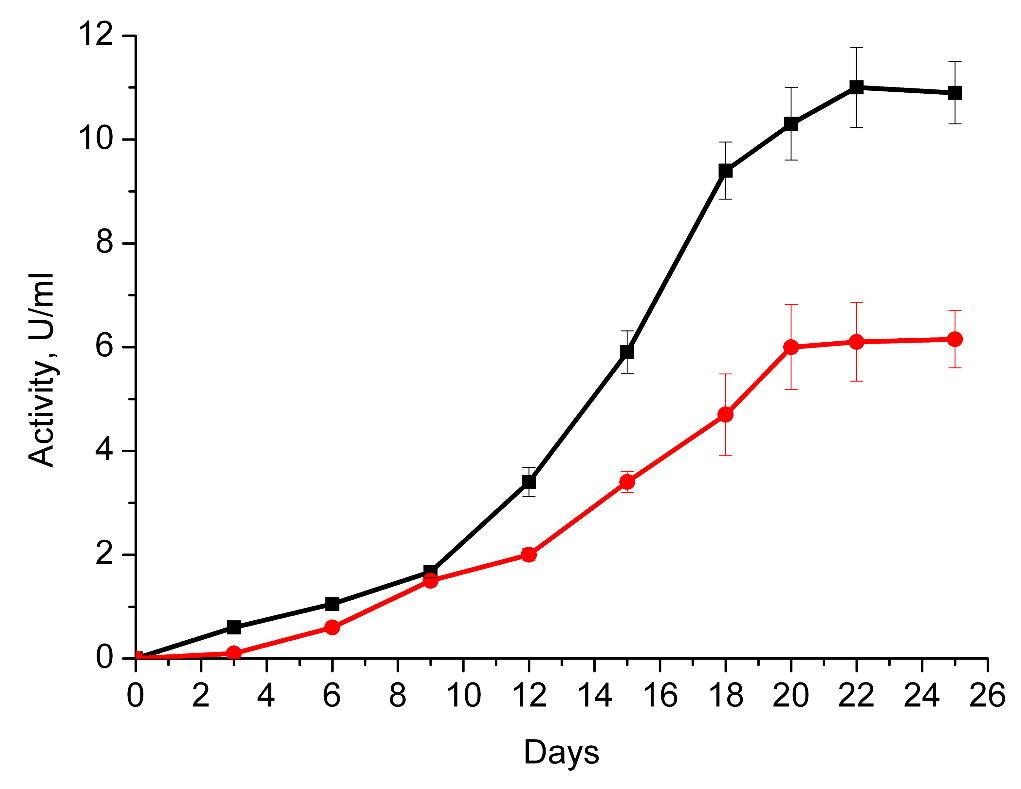
**

**Figure E. UV-Vis spectra of JnL.**


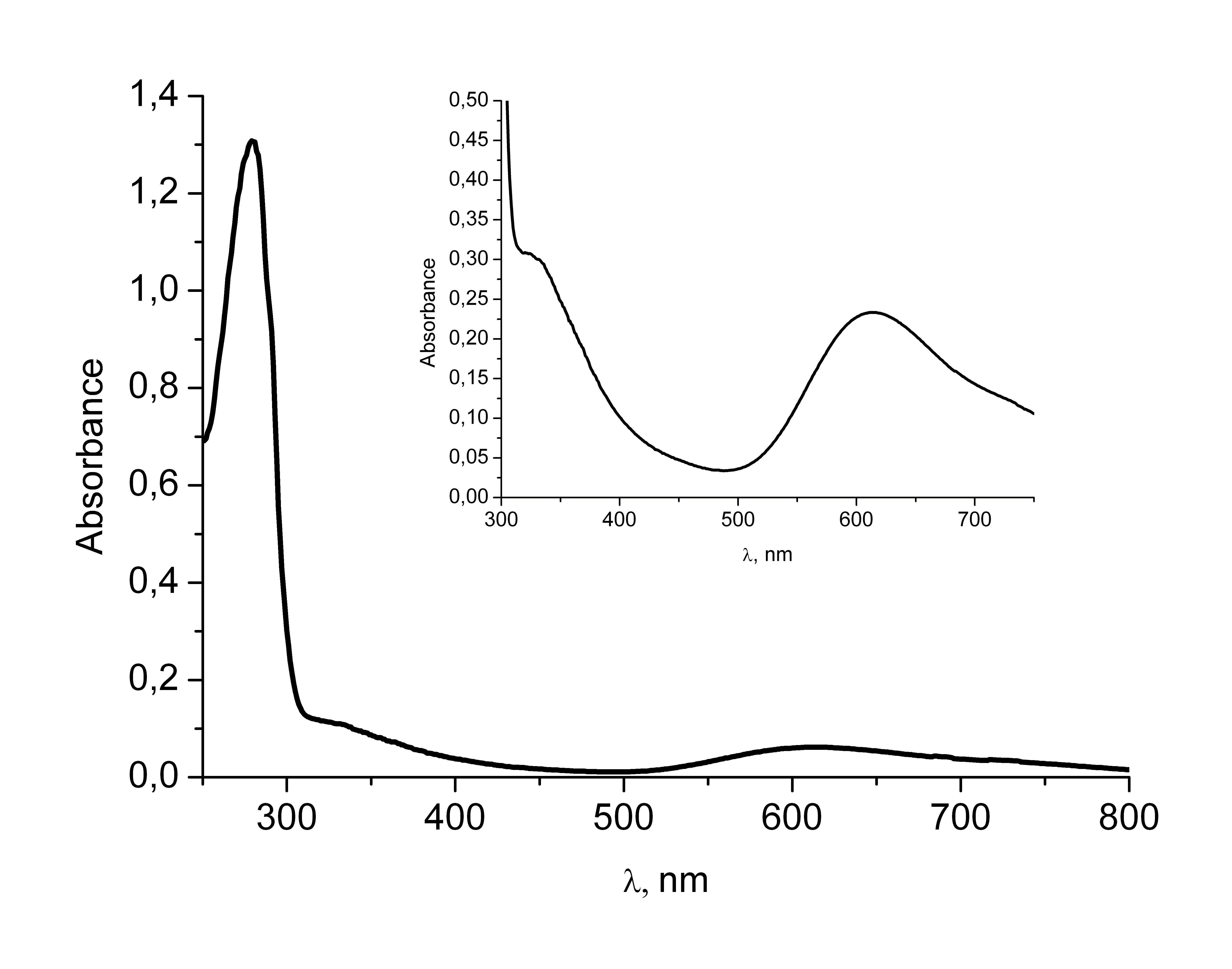


**Figure F. Redox titrations of JnL (A) and SbL (B).**





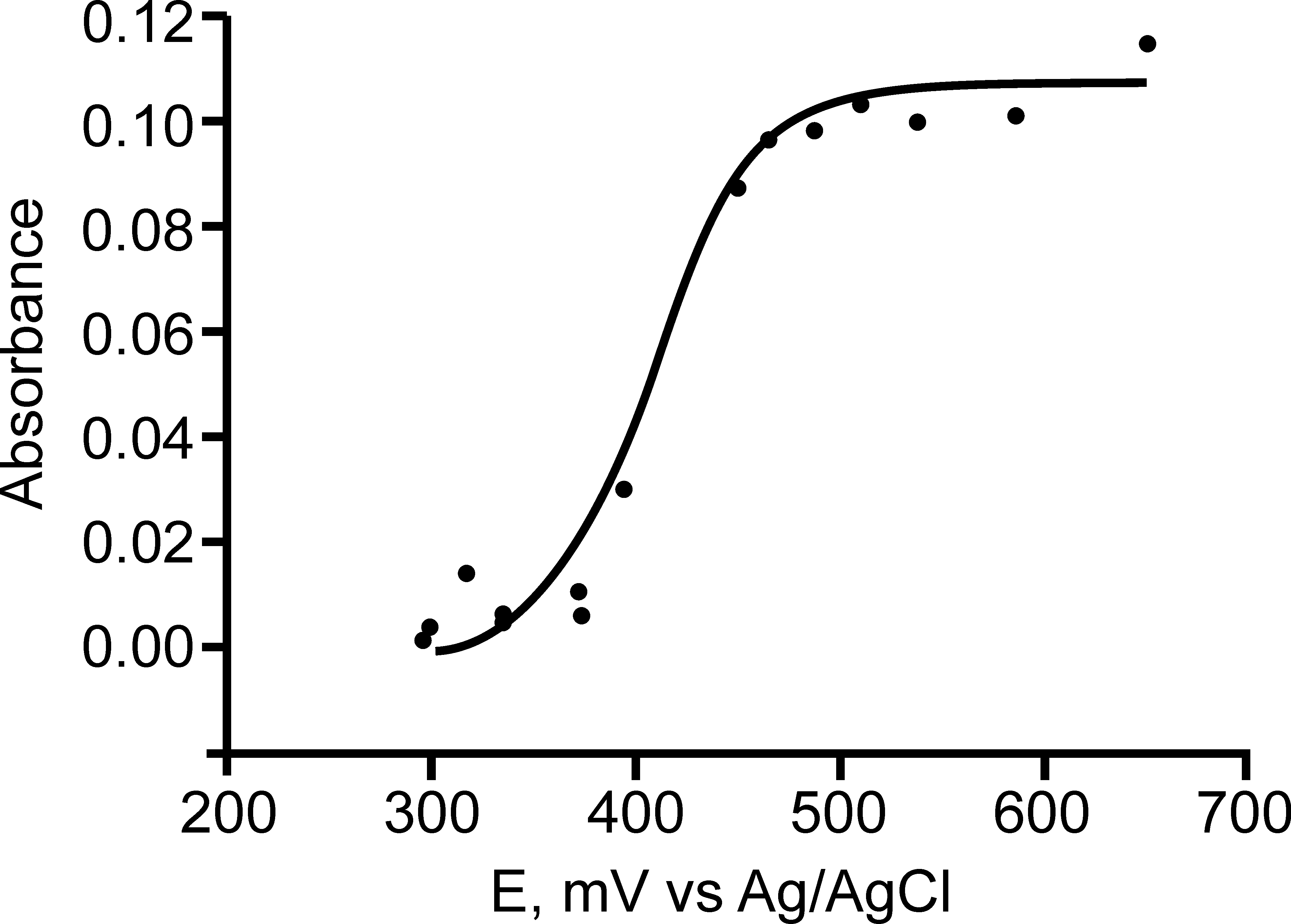


A B
